# Inequalities by energy sources: An assessment of environmental quality

**DOI:** 10.1371/journal.pone.0230503

**Published:** 2020-03-20

**Authors:** Xing Yao, Rizwana Yasmeen, Ihtsham Ul Haq Padda, Wasi Ul Hassan Shah, Muhammad Abdul Kamal

**Affiliations:** 1 School of International Business, Southwestern University of Finance and Economics, Chengdu, China; 2 Department of Economics, Federal Urdu University of Arts, Science and Technology, Islamabad, Pakistan; 3 School of Statistics, Southwestern University of Finance and Economics, Chengdu, China; 4 Department of Economics AWKUM, Mardan, Pakistan; School of Economics, Xiamen University, CHINA

## Abstract

Energy demand is rising day by day, driven mainly by the development of countries. At the same time, uneven economic growth in countries is the prime cause of inequality in energy consumption. Keeping in view the worth of energy in the growth process, this study quantifies the impact of energy inequalities and trade on environmental quality over the period 1995–2018 for 57 countries. The Theil approach is used to quantify inter-and intra-regional disparities in five energy sources; oil, coal, natural gas hydroelectricity, and renewable energy. The results show that North America has the highest oil consumption inequality between the regions while East Asia & Pacific has the highest index value within the regions. Coal consumption inequality is declining in North America, but not in East Asia and the Pacific. Europe & Central Asia, and North America have the highest inequalities in natural gas consumption between the regions. Inequality is shrinking in hydropower consumption between the regions, however, such trend has not loomed within the regions. Europe & Central Asia and East Asia & Pacific have major renewable consumption inequalities within the regions. Generally, there is a decreasing temporal trend in energy consumption inequalities of all energy sources. The GMM technique is applied to investigate the impact of energy inequalities and trade openness on environmental quality. The results reveal that energy inequalities degrade environmental quality. Moreover, trade has a positive impact on environmental quality. However, democratic countries can be advantageous to improve the environmental quality. The study implies that countries should take actions to reduce energy inequalities within and between the regions. Specialization in production through trade can also be an option for improvement in the environment.

## 1. Introduction

**Fig 1:**
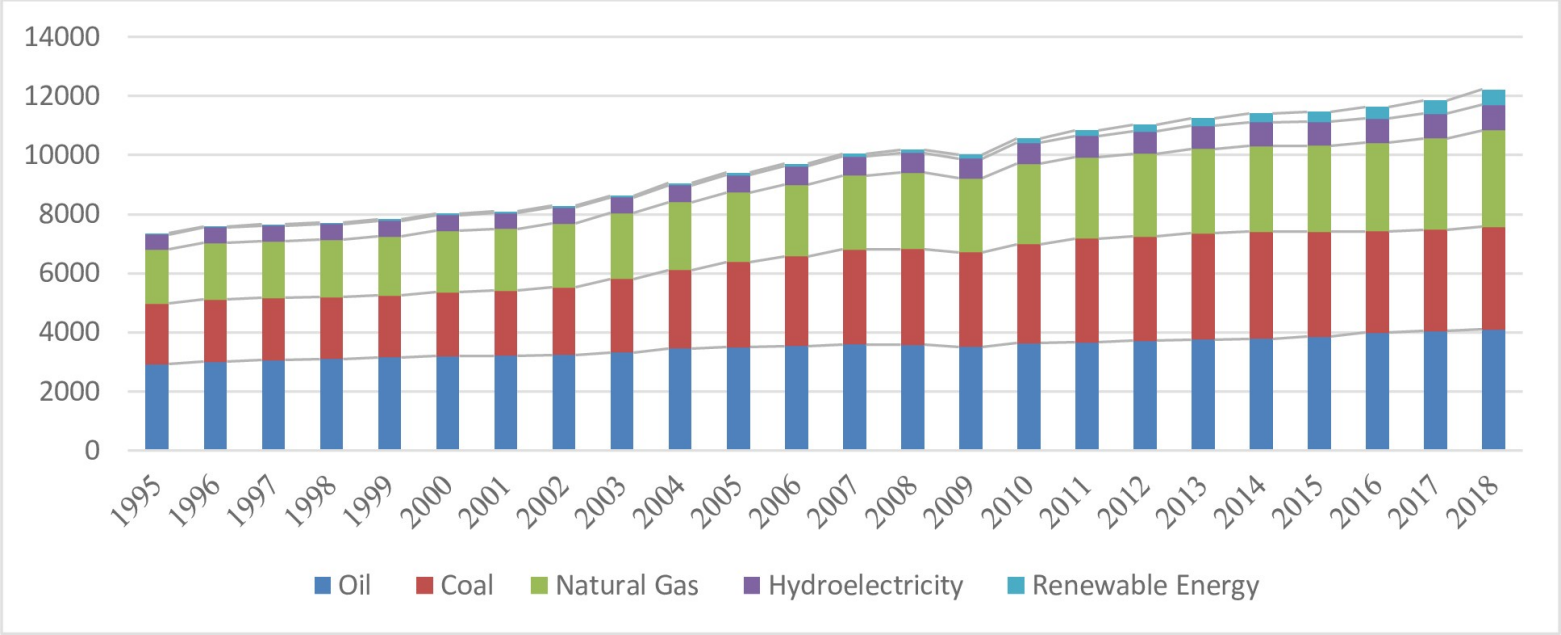
World energy consumption pattern by sources in million tonnes (1995–2018). Compiled from World Energy Outlook (2018–2019).

Energy is one of the most important inputs for economic growth and development [1]. It is the indispensable need of each sector of the economy, such as household, industry, and agriculture [2, 3]. On the contrary, a decrease in energy consumption can affect the development process directly or indirectly [4]. China is an excellent example that started mega energy projects under the umbrella of the Belt & Road initiative (BRI), to meet an adequate energy level. On average, the surge in world energy demand is steadily increasing due to economic development and population growth [5]. According to world energy statistics 2019 [5], oil consumption rose by 1.4 million barrels per day (Mb/d), driven mainly by the growth of the developing world including China and India. The US, however, was the biggest outlier in driving demand for oil, which grew by 0.5Mb/d in 2018. Coal is the second major source of energy globally, and its demand increased by 1.4% in 2018, twice over ten years ago. While coal is China's largest source of energy, environmental policies are now focused on transferring coal to gas for domestic needs to protect air quality. Growth in the production and consumption of natural gas in 2018 was above 5%, the highest in the last 30 years, accounting for 40% of global demand in natural gas consumption. The share of renewable energy consumption including wind, solar, hydropower continued to grow rapidly, but the share of mix fuels remained unchanged in 2018 [6]. Fig 1 demonstrates year-wise trends in energy consumption of the world. It depicts that oil, coal and natural gas are the main sources of energy, while the use of hydroelectricity and renewable energy are comparatively low [6]. In a nutshell, the share of renewable resources in energy demand is rising gradually. However, the use of energy has two divergent effects on growth and the environment. Rapid economic growth is subject to energy use [7, 8], which ultimately adversely affects environmental quality [9, 10]. More than 80% of the energy supply is based on coal, oil, and gas which release enormous quantities of carbon gas into the air [11]. Thus, inequalities in energy use both within and between regions are at times starker, which stems from differences in incomes, production and consumption, and lifestyles [12]. The use of energy inequalities is an inherent feature of the distribution of energy resources and its impact on climate change. This arises one fundamental question of how inequalities in energy use by different sources affect the environment. One plausible way is to look at the countries’ energy source system. For instance, the UK is now the low emitter comparatively due to its access to natural gas from the North Sea while France largely relies on nuclear power for electricity production [13]. Globally, developed countries have more access to clean energy sources such as nuclear, wind and solar. However, developing countries are still lagging behind in gaining access to advanced energy technologies [14].

On the other side, unequal consumption patterns throughout the world also posing severe environmental challenges. Further, the lack of modern energy sources limits productive opportunities that have a negative impact on human health and welfare, who are often exposed to detrimental emissions. Moreover, the poor segment of the population always depends on highly-polluting forms of energy to meet their basic needs which ultimately poses a significant threat to the environment [12]. Most of the global carbon emission is associated with human lifestyles (IPCC 2001, 2014) [15, 16] and it varies across the world, consequently damaging the global eco-system. Nearly 90 percent of the world’s commercial energy is derived from non-renewable energy sources, while the proportion of renewable energy such as hydro-power & nuclear energy is marginal [17].Therefore, energy inequalities are often reflected as inequalities in income and other developmental dimensions which may contribute directly or indirectly to environmental degradation. Hence, there is a dire need to switch from high to low carbon releasing energy systems [17]. Indeed, reliance on renewable energy sources is growing steadily but its consumption share is nominal (see Fig 1). Despite the improvements in the power sector the growth in energy demand continues to increase significantly. Particularly as developing countries are pursuing industrialization and trade for rapid development that causes to increase the atmospheric level of carbon emission. [18].

Trade is an important economic aspect that affects the environment by accelerating economic development [19, 20]. Trade activities are linked with higher energy use, particularly in the manufacturing of industrial goods and transportation of final products. A plethora of studies have examined the environment-trade nexus; however, the results are mixed and inconsistent. For instance, Akin [21] argues that trade mitigates the environmental problems while others using various parameters with trade conclude positive effect of trade on the environment [22–26]. Likewise, Tiwari et al. and Chebbi et al. [27, 28] reported positive relationship between trade and the environment. In the global value chain scenario, Yasmeen et al. [29] found that at the earlier stage of development trade harms the environment but at the later stage it improves the environmental system by adopting clean technologies. Another study conducted by Wu and Wang [30] determines the drivers of emissions embodied in provincial trade and argues that final demand and carbon emission intensity are leading elements for emissions embodied in trade. Furthermore, the structure of trade is also important reason for emission encompassed. Nevertheless, Antweiler et al. [31] decomposed the positive and negative impact of trade on the environment into three possible ways: (i) the scale effect, (ii) technique effect, and (iii) composition effect. Under the scale effect, trade stimulates development process via production and the use of energy, which ultimately damage the environmental efficiency [32, 33]. While, in the second phase of the development, the state engages in cleaner production because of sophisticated technologies with less/environmental friendly energy consumption which in turn improves environmental efficiency [34] called the technique effect. The composition effect arises when the share of emission-intensive goods in the production processes decreases [10, 31, 35]. Above and beyond, effective trade policy on energy consumption is necessary to attain sustainable growth with a clean environment. Trade can yield efficiency by access to eco-friendly technologies and via modern production methods which can reduce energy inequalities [35].

Though trade activities are inevitable without the effective use of the energy and its expansion reshapes the demand for energy due to a surge in the production rate. Developing countries are still tied up with obsolete energy-intensive production methods. Whereas, trade allows developing economies to access advanced energy-saving technologies and reduce energy inequalities between trading countries. Thereby, energy inequalities are also an important mechanism for predicting energy consumption patterns along with trade. Moreover, diverse types of energy sources have different effects on the environment. Hence, to achieve concrete results, it is imperative to conduct further investigation into energy inequalities and environmental quality nexus with different energy sources. Henceforth, energy inequality in its use cannot be overlooked in the process of environmental degradation. Thus the aim of this study is to unravel the disparities of energy consumption in renewable and non-renewable sources of energy. Each source of energy has its own energy consumption pattern that can differ in its inequalities and environmental impacts. Therefore, considering one source of energy is not sufficient to comprehend the entire consumption pattern of the energy sector. This study contributes to the growing literature on energy in many ways. First, it has selected five main energy sources; oil, coal, natural gasses, hydro, and renewable energy to explore energy consumption patterns and disparities. It also spotlights on non-renewable (oil, coal, natural gas) and renewable (hydro and other renewables) energy disparities. To the best of our knowledge, this paper is the first of its nature to investigate disparities of energy consumption in renewable and non-renewable sources of energy. Second, the trade sector heavily relies on energy as an input in the manufacturing of industrial goods and the transportation of products. Trade openness can, therefore, be an important factor in reducing inequalities by opening the door to more equal opportunities, especially for developing countries towards energy sources and technologies. Thus, this study also invokes the role of trade openness in the presence of energy inequalities. Additionally, we incorporate the role of political regimes which will be helpful in giving insightful implications to policymakers. Finally, the results could vary across the countries; thus the estimated functions might suffer from the problem of parameter heterogeneity. For the sake of robustness and to tackle the issue of parameter heterogeneity in estimated functions, estimation results are obtained both at global and regional levels. Moreover, this study also analyzes how energy inequality in inter- and intra- regions affects the environment. This enables us to recommend insightful measures for controlling energy inequalities among the regions. Energy disparities are calculated via cross-entropy (Theil's index) in five energy sources for the world and six regions.

## 2. Data and research methods

### 2.1 Theil inequality index and econometric approach

The Theil inequality index [36] based on “Cross-Entropy” function is used to quantify the consumption disparities in oil, coal, natural gas, hydropower, and renewable energy. Theil index computes the disparities of random variables between two sets of distributions [37]. This index measures the information inequality between two probability distributions [36]. Moreover, “Theil’s index” is more convenient and gives a more accurate picture of inequality within and between defined population groups [38]. Because it allows decomposing difference into the parts i.e. one due to inequality within regions and other is due to dissimilarities between regions. It aggregates the inequalities at each level/hierarchy of data as the final value of the Theil's Index is made of two components such as between the region and within the region. Upon this, it is a better measurement of regional energy consumption inequalities than others.

To extract the information inequality in the distribution cross-entropy (CE), the following equation can be used:

$${CE}_{t}=\sum_{i=1}^{N}c_{it}\;ln\left(\frac{c_{it}}{d_{it}}\right)$$ (1)

Where, *c*_*it*_ is the prior probability of an event occurred, and *d*_*it*_ is the subsequent probability from unexpected information for “ith” country in the year “t”; i = 1,2,……N, and t represents time (annual) t = 1,2……t. Precisely, since ”*c*_*it*_” is the energy consumption by different sources and “*d*_*it*_” is the population of “ith” country, then energy consumption share by source (*E*_*s*_) can be computed as follows:

$$e_{s,t}=\frac{E_{s,it}}{\sum_{i=1}^{N}E_{s,it}}$$ (2)

Where, *E*_*s*,*it*_ is the energy consumption of “s” source of the country “i” in the year “t”, while, $\sum_{i=1}^{N}E_{s,it}$ is the total energy consumption of “s” source of all “N” countries at time “t”. Just as, the population share of a country at year “t” can be computed as follows:

$$p_{t}=\frac{P_{it}}{\sum_{i=1}^{N}P_{it}}$$ (3)

Where, *P*_*it*_ is the population of the country “i” in year “t”, while, $\sum_{i=1}^{N}P_{it}$ is the total population of all “N” countries at time “t”. The cross-entropy (CE) for energy consumption inequality by energy source can be calculated as follows:

$${CE}_{t}=\sum_{i=1}^{N}e_{s,it}\;ln\left(\frac{e_{s,it}}{p_{it}}\right)$$ (4)

Eq (4) is used to measure energy consumption inequality in oil coal, natural gas, hydropower, and renewable energy. This index quantifies whether the consumption of the energy among the economies is diverging or converging. The *CE*_*t*_ reduces if energy consumption inequality decreases over time, in contrast, vice versa. The total energy consumption inequality (*At*_*s*_) for different energy sources can be computed through within and between regions inequalities. Suppose a world grouped into a region (“r”) and it consists of many countries that have different energy consumption patterns in different sources. If a country situated in “r” region for = 1995, 1996,…,2018 then inequalities index of between-regions (“*bt*”) and within- regions (“*wt*”) for “s” energy- source can be estimated as:

$${bt}_{s,t}=\sum_{r=1}^{R}E_{s,rt}\;ln\left(\frac{E_{s,rt}}{p_{it}}\right)$$ (5)

$${wt}_{s,t}=\sum_{i=1}^{r}\frac{e_{s,it}}{E_{s,rt}}\;ln\left(\frac{\frac{e_{s,it}}{E_{s,rt}}}{\frac{p_{it}}{P_{rt}}}\right)$$ (6)

Where, energy consumption share in “s” source of region “r” is defined as $E_{s,rt}=\sum_{i=1}^{r}e_{s,it}$, and share of the population is as $P_{rt}=\sum_{i=1}^{r}p_{it}$. The “*At*_*s*_” is the sum of “*bt*_*s*,*t*_” and “*wt*_*s*,*t*_” inequalities that described in the following ways:

$${At}_{s}={bt}_{s,t}+\sum_{r=1}^{R}E_{s,rt}\times {wt}_{s,t}$$ (7)

Between–regional inequality “*bt*_*s*,*t*_” calculated the energy consumption inequality that exists in different energy sources, while “*wt*_*s*,*t*_” measures the energy consumption inequality in different energy sources within countries in region “r”.

Energy consumption is an important pillar for the sectoral growth of economy including household, industry, transportation, agriculture and others those consuming energy resources at an unsustainable rate. This instability in energy consumption increases the potential for resource-based geopolitical conflicts that prevent nations to develop collectively to global climate threats. Moreover, it has been acknowledged by the environmental researchers that greenhouse gases produced by human activities have detrimental impacts on the global environment. A nation with unhealthy energy consumption patterns due to lack of cleaner sources poses a great threat indirectly towards the environment that cannot be ignored in the coming years. On the other side, trade can open doors to access advanced energy resources. Given the importance of energy consumption inequalities the empirical model is composed following Gozgor; Hasson and Masih; Pascual Sáez et al.; Hafeez et al. [22, 23, 39, 40] as:

$${EQ}_{it}={\partial_{1}EQ}_{i,t-1}+{\partial_{2}GDP}_{it}+{\partial_{3}GDPS}_{it}+\partial_{4}{ECS}_{it}{{+\partial }_{5}TO}_{it}+\partial_{6}{PRG}_{it}+\varepsilon_{it}$$ (8)

Environmental quality is signified by *EQ*_*it*_. *ECS*_*it*_ is the set of energy inequality in oil, coal, gas, hydropower and renewable consumption (BECS is between energy consumption inequality, WECS is within energy consumption inequality, TECS is total energy consumption inequality) are expected to be positive. TO is the trade openness, which can be either positive or negative. While composing the premise for examining the impact of energy consumption inequalities on environmental quality, the development process and prevailing political situation can also affect the level of carbon emission. Therefore, consistent with the literature, [10, 22] this study includes the GDP (predicted to be positive) and SGDP (anticipated to be negative) to quantify the impact of development on environmental quality. The countries’ institutions have been argued to benefit countries' commitment to improve the environmental quality by adopting stricter environmental policies, and curb carbon dioxide emission [41, 42, 43]. Moreover, democratic institutions are expected to be stronger enough to in climate change mitigation than non-democratic regimes. The democratic countries are under pressure of their voters to take actions for improvement in the energy efficiency and environmental quality. Thus to capture the political regimes (PRG) effect the political regime index is used which is classified as closed autocracy, electoral autocracy, electoral democracy, liberal democracy. The empirical estimates will be helpful in giving insightful implications to policymakers in the light of energy inequalities and trade. Moreover, the application of different energy sources will elaborate on the inequality situation in energy consumption substantially.

### 2.2 Generalized Methods of Moments (GMM)

In the present study, the dynamic panel data model is estimated. To evaluate the significant impact of the concerned explanatory variables on the environmental quality, we use the Blundell and Bond system GMM methodology. The GMM is the most popular estimation technique if time span (T) is less than cross-sections (N) Arellano and Bond [44]. The choice of system GMM is justified on the basis that if the dependent variable is persistent to a random walk then difference GMM performs poorly, as past values are vague about future changes. So, higher-order lags of the regressors are weak instruments for the differenced variables [45]. In such case, system GMM is the best choice Blundell and Bond [46]. Secondly, fixed effects estimator is biased in the presence of the lagged dependent variable and it also accounts for possible endogeneity issues. Moreover, if the difference GMM estimates lie below or close to fixed effects, this will be biased downward, and consequently, system GMM would be efficient. Additionally, GMM estimator, in the absence of MLE, can be used as an alternative to other methods. The beauty of both difference and system GMM methods are the use of the instruments which are valid based on the assumption that the disturbance terms are truly independent and are serially uncorrelated. Therefore, the Arellano-Bond test, checks for serial correlation in the residuals by testing the residuals in the differenced equations for serial correlation. However, the first-order serial correlation is to be expected and therefore the key test is to check for second-order serial correlation which should not be rejected the null hypothesis of no second-order serial correlation. Moreover, the joint validity of the instruments can be verified by running the Sargan/Hansen test.

### 2.3 Data

In the first instance, global analysis has been performed to identify the inequalities of energy consumption in oil, coal, natural gas, hydroelectricity, and renewable energy. Then, sample countries have been classified for regional comparison, based on World Bank data definition. Due to the unavailability of data, the Middle East & North African region is excluded while we quantified the inequality in renewable energy consumption. Further, due to the data limitation, we have selected overall 57 countries for empirical analysis over the period 1995–2018. The detail of sampled countries is provided in Table A1 in S1 Appendix. The data for CO2, oil, coal, natural gas, hydroelectricity, and renewable energy (including wind, geothermal, solar, biomass) are extracted from the World Energy Outlook [6]. While the data of population and trade are taken from the World Development Indicators [47]. The data description in detail is mentioned in Table 1.

**Table 1:** Data description.

Variables	Symbolization	Measures	Data source
Environmental quality	EQ	CO2 emission million tonnes	World Energy outlook
Population	POP	Population, total	World Development Indicators
Oil	OC	Consumption in Million tonnes	World Energy outlook
Coal	CC	Consumption in Million tonnes	World Energy outlook
Naturel gas	NC	Consumption in Million tonnes	World Energy outlook
Hydroelectricity	HC	Consumption in Million tonnes	World Energy outlook
Renewable energy	RC	Consumption in Million tonnes	World Energy outlook
Trade Openness	TO	Trade percentage of GDP	World Development Indicators
Goss domestic product	GDP		World Development Indicators
	GDPS		
Political Regimes	PRG	political regime classification as (Closed autocracy = 0; Electoral autocracy = 1; Electoral democracy = 2; and Liberal democracy = 3)	Our World in Data

### 2.4 Panel correlation matrix of energy inequalities by source

The correlation statistics are reported in Table 2. The environmental quality (EQ) is positively correlated with between (BECS), within (WECS), and total (TECS) energy inequality for oil, coal, and natural gas. It demonstrates that inequality in energy sources degrades environmental quality by rising CO2 emissions. However, hydropower source is negatively related to within energy inequality and the environmental quality. There is also a negative association between and within renewable energy inequality and EQ. This negative direction reveals that cleaner sources of energy are key to reduce CO2 emissions, even though inequality exists in its use. The variable of trade openness is negatively correlated with environmental degradation. While, at the initial stage of development, income (GDP) decrease the environmental quality (EQ), while, in the second stage of the development it improves as the correlation between SGDP and EQ is negative. In addition, trade decreased the energy inequality between and within regions. Moreover, it is also effective in reducing total energy inequalities. In a nutshell, trade opens door to more equal opportunities for developing countries towards energy sources and technologies. As per expectations political regime positively contributes to improve the environmental quality.

**Table 2:** Panel correlation matrix of energy inequalities by source.

Variables	EQ	GDP	GDPS	BECS	WECS	TECS	TO	PRG
**EQ**	1							
**GDP**	0.286	1						
**GDPS**	-0.239	0.941	1					
**BECS**	0.008	0.075	-0.073	1				
**WECS**	0.009	0.083	-0.080	0.846	1			
**TECS**	0.008	0.081	-0.078	0.987	0.915	1		
**TO**	-0.398	-0.183	-0.164	-0.062	-0.112	-0.077	1	
**PGR**	-0.014	0.115	0.085	0.021	0.010	0.020	-0.026	1
**Panel _ correlation for Coal**								
**EQ**	1							
**GDP**	0.286	1						
**GDPS**	-0.238	0.941	1					
**BECS**	0.009	0.077	-0.076	1				
**WECS**	0.001	0.001	0.007	0.3164	1			
**TECS**	0.008	0.056	-0.058	0.8828	0.605	1		
**TO**	-0.398	-0.183	-0.164	-0.0731	--0.047	-0.052	1	
**PRG**	-0.013	-0.115	-0.085	-0.0236	-0.024	-0.024	-0.026	1
**Panel _ correlation for Natural gas**								
**EQ**	1							
**GDP**	0.286	1						
**GDPS**	-0.239	0.941	1					
**BECS**	0.007	0.081	0.080	1				
**WECS**	0.009	0.080	0.078	0.884	1			
**TECS**	0.008	0.078	0.076	0.905	0.984	1		
**TO**	0.398	0.183	0.164	-0.076	-0.083	-0.065	1	
**PRG**	-0.014	0.115	0.085	0.017	0.020	0.023	-0.026	1
**Panel _ correlation for hydroelectricity**								
**EQ**	1							
**GDP**	0.286	1						
**GDPS**	-0.239	0.941	1					
**BECS**	-0.009	0.083	-0.081	1				
**WECS**	0.005	0.018	-0.019	0.184	1			
**TECS**	0.009	0.080	-0.078	0.959	0.440	1		
**TO**	-0.398	-0.183	-0.164	-0.091	-0.023	-0.084	1	
**PRG**	-0.014	-0.115	-0.085	-0.019	-0.008	0.021	-0.026	1
**Panel _ correlation for renewable**								
**EQ**	1							
**GDP**	0.286	1						
**GDPS**	-0.239	0.941	1					
**BECS**	-0.007	-0.081	-0.080	1				
**WECS**	-0.009	0.080	-0.078	0.884	1			
**TECS**	0.008	0.078	-0.076	0.905	0.984	1		
**TO**	-0.398	-0.183	-0.164	-0.076	-0.083	-0.065	1	
**PRG**	-0.014	-0.115	-0.085	-0.017	-0.020	-0.023	-0.026	1

## 3. Estimates of energy inequality

**Fig 2:**
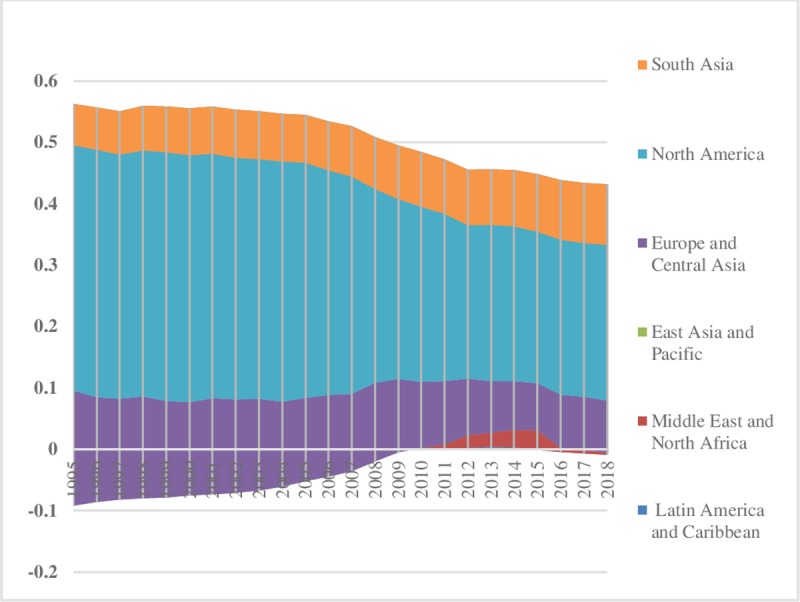
Pattern of inequality between the regions for oil.

**Fig 3:**
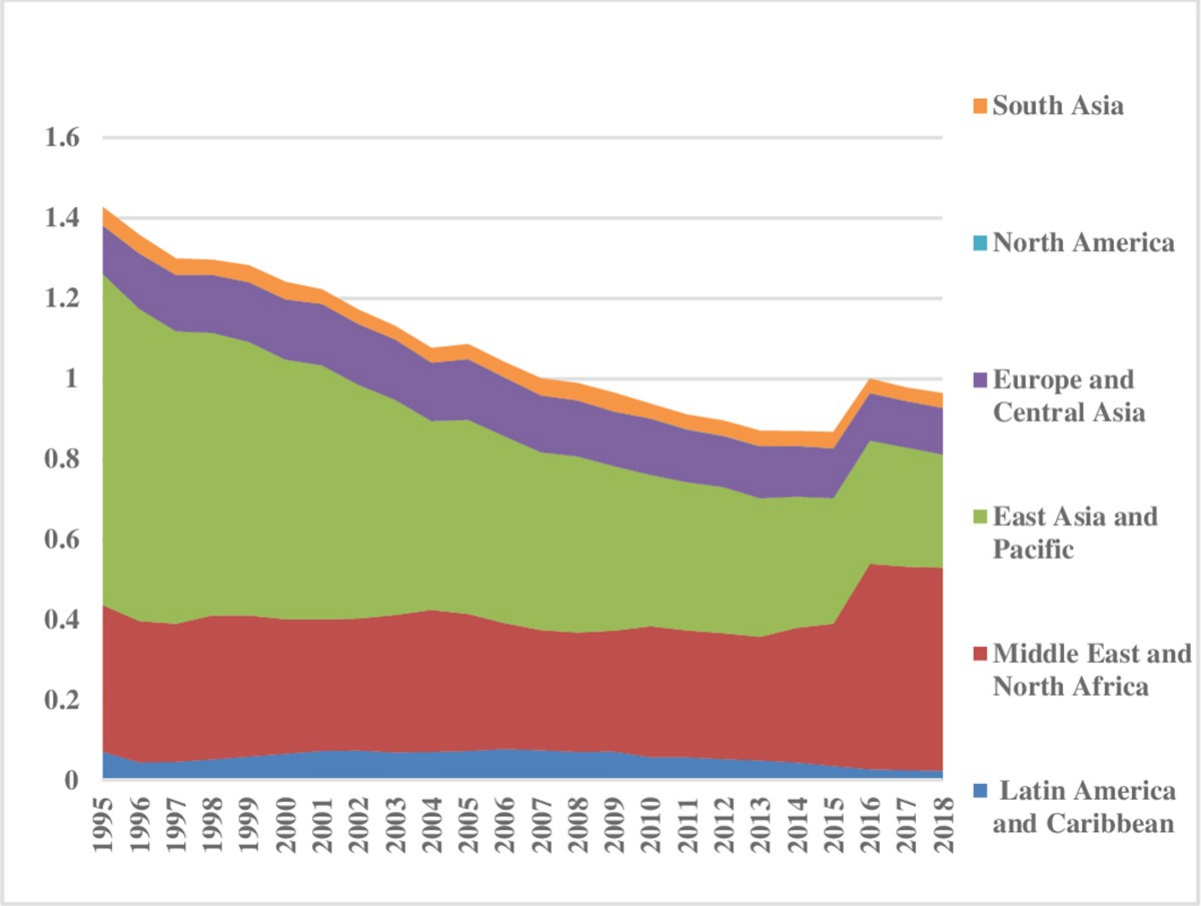
Pattern of inequality within the regions for oil.

**Fig 4:**
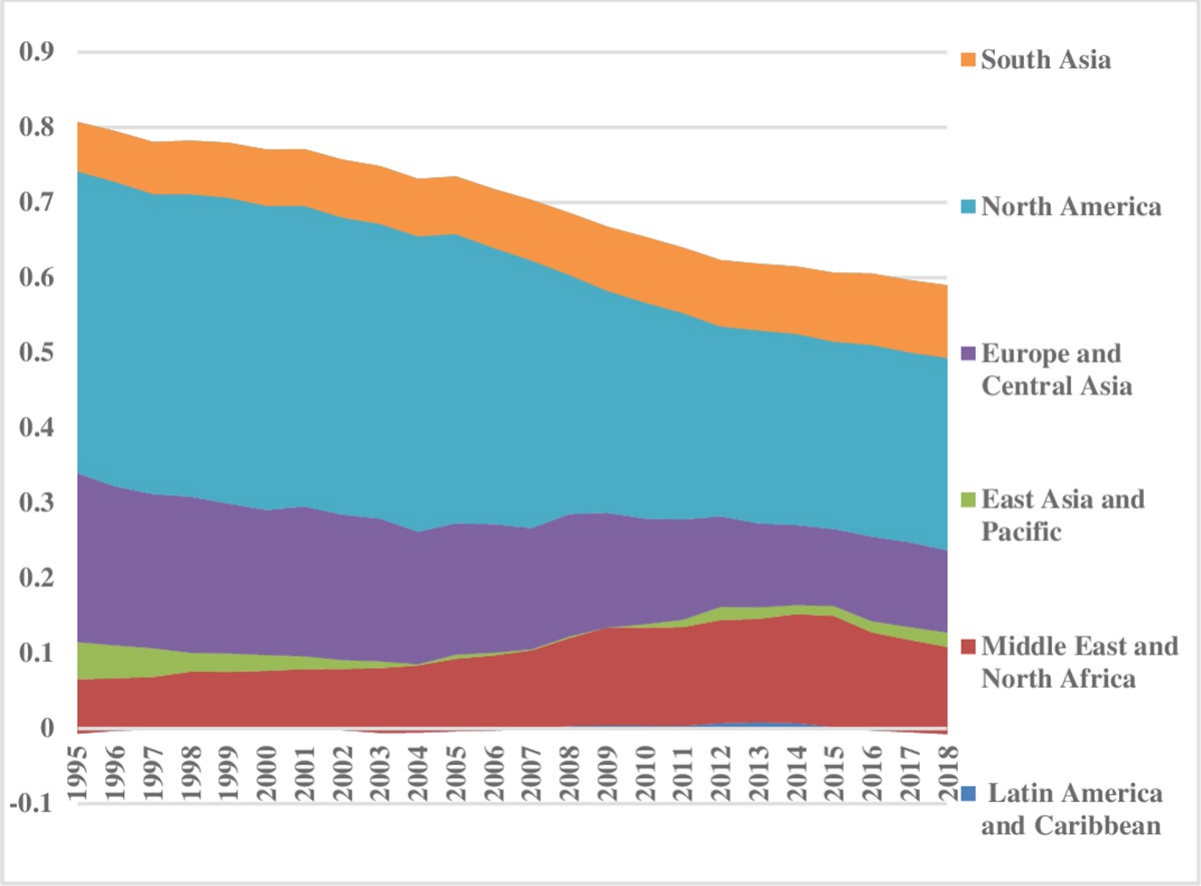
Pattern of total inequality for oil.

The world energy consumption disparities are computed by using the model presented in Eq (4). The oil energy consumption inequality graphs depict that the index of oil energy inequality has decreased gradually over the sample period (1995–2018). The results of Fig 2 show that South Asia, East Asia & Pacific, Latin America & Caribbean, Middle East & North Africa have less energy inequality in oil consumption between the regions. Meanwhile, North America, and Europe & Central Asia are showing major and minor disparities in oil energy consumption, respectively. These energy inequalities in oil consumption indicate that every region has a different growth level. Moreover, economies differ in production methods, market size, industrial growth, and weather conditions. Fig 3 depicts the prevailing scenario of oil energy inequality within regions. It has decreased considerably over time in East Asia & Pacific while in the recent past it has increased in the Middle East & North Africa. Europe & Central Asian region has an average level of discrepancy among the regions while Latin America & Caribbean and South Asia show very fewer differences in oil consumption. North America also has minor inequality within regions. The energy inequalities within regions show a declining trend in Europe & Central Asia, South Asia, and East Asia & Pacific. Total energy inequality in oil consumption is shown in Fig 4. In the sample period the highest disparity remained in North America which has decreased over the time. However, total inequality has risen over the time in South Asia and Middle East & North Africa regions. East Asia & pacific indicates little disparity in total oil consumption inequality. In addition, North America has significant inequalities while East Asia & Pacific and South Asia have less intensity in total oil consumption inequalities.

**Fig 5:**
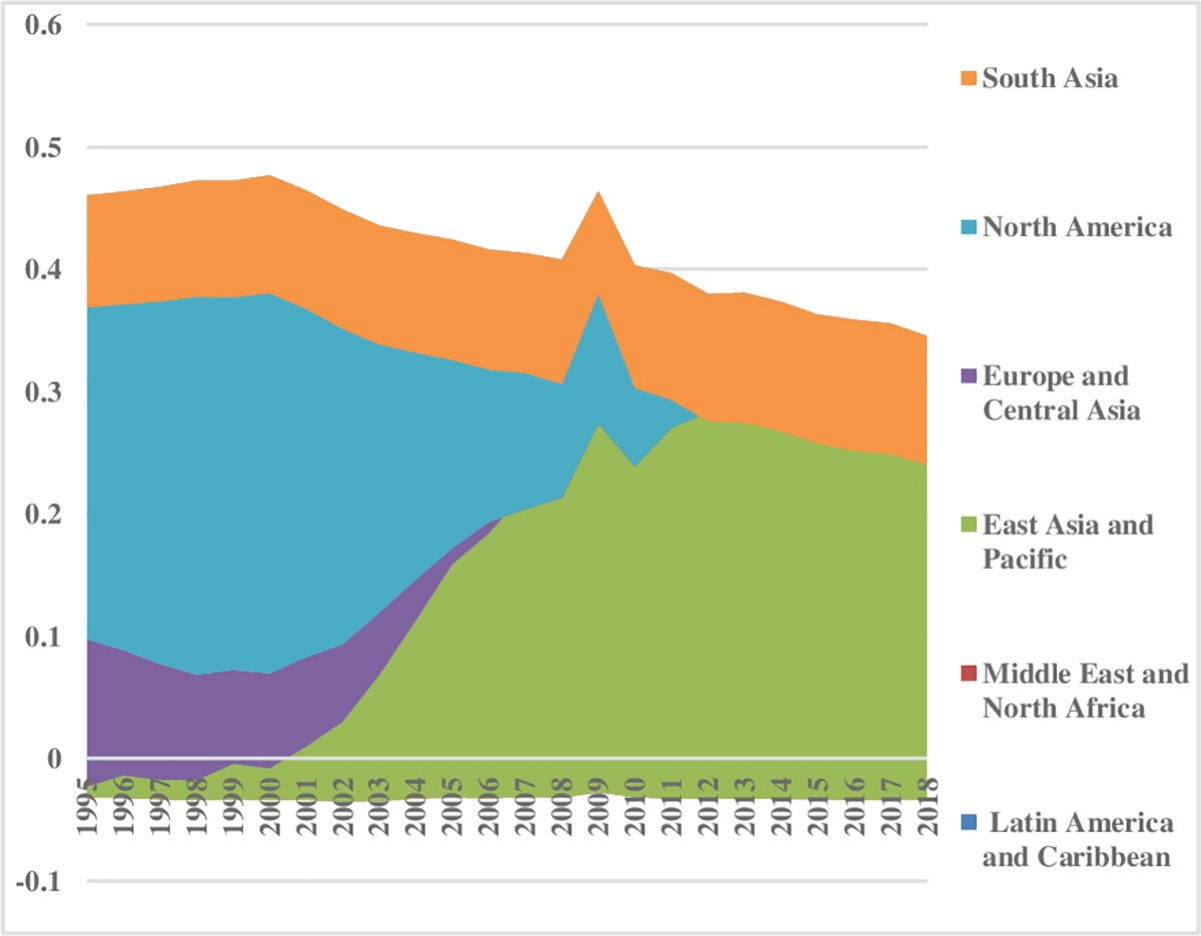
Pattern of inequality between the regions for coal.

**Fig 6:**
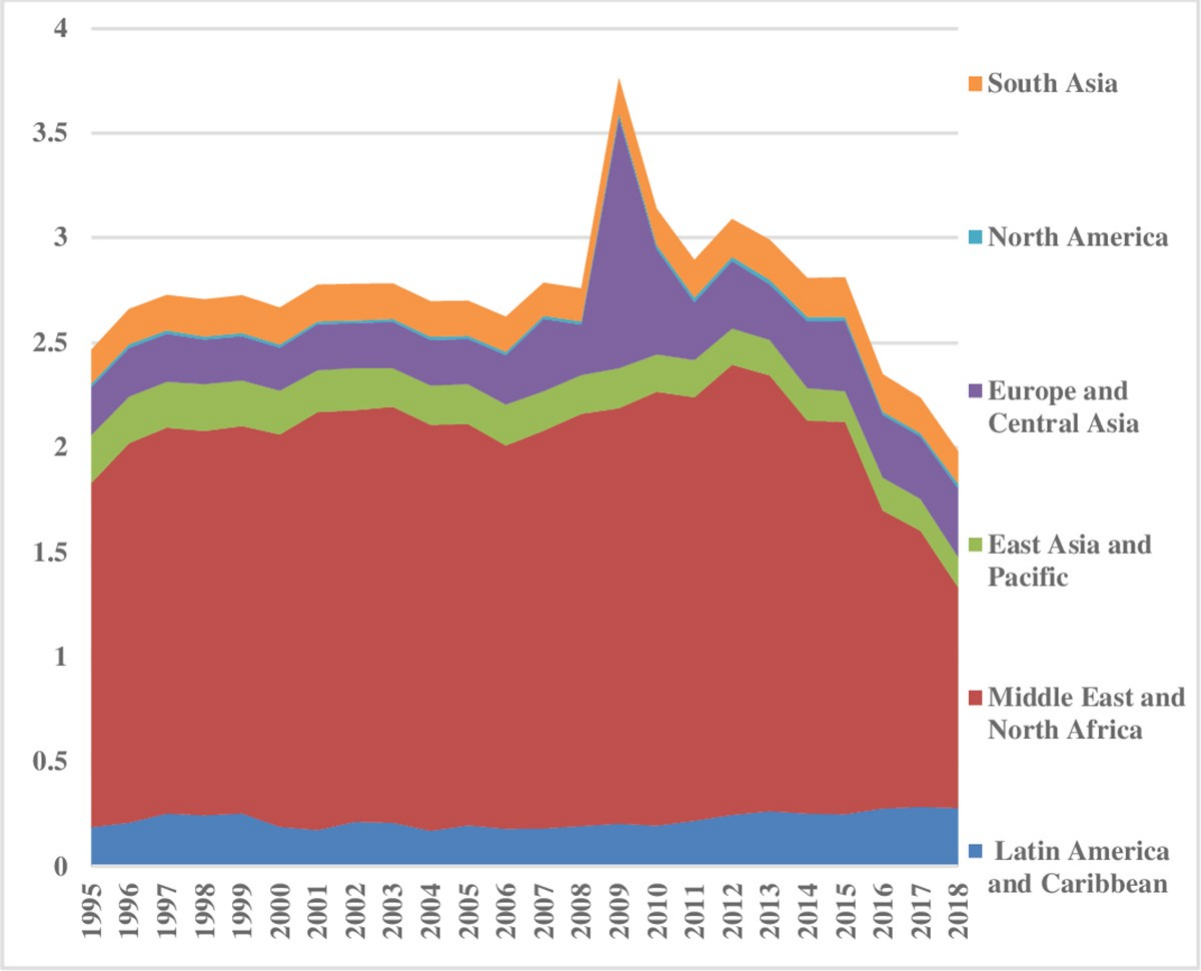
Pattern of inequality within the regions for coal.

**Fig 7:**
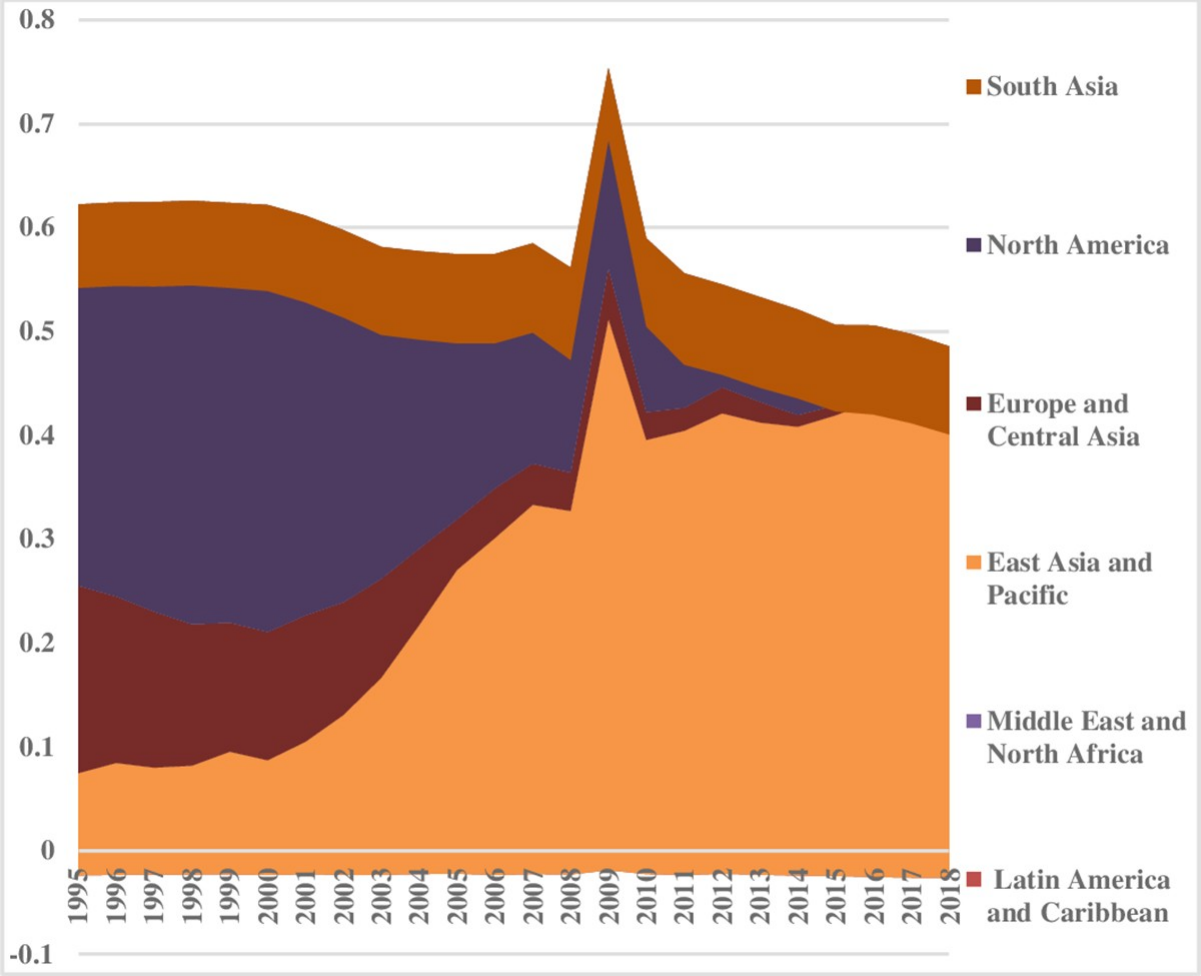
Pattern of total inequality for coal.

Fig 5 illustrates the coal energy inequality between the regions. The energy inequality in levels of coal consumption between regions stayed lower in the Middle East & North Africa, Latin America & Caribbean, and South Asia. It has decreased significantly in North America. East Asia & Pacific shows minor disparities from1995 to 2002, afterward a rising trend is evident. This uprising tendency in East Asia & pacific is possibly due to the expansion of China’s economy and population size. Moreover, coal is a major source of energy in China. Europe & Central Asia has the lowest disparities between the regions. The pattern of coal consumption inequality within regions is shown in Fig 6 which remained almost constant. However, the disparities are at the highest level in Middle East & North Africa compared to others. The index depicted comparatively less inequality within North America and Latin America & Caribbean regions. Total coal consumption inequalities are demonstrated in Fig 7. The total inequality has declined substantially in North America and Europe & Central Asia while the increasing trend is observed in East Asia & Pacific.

**Fig 8:**
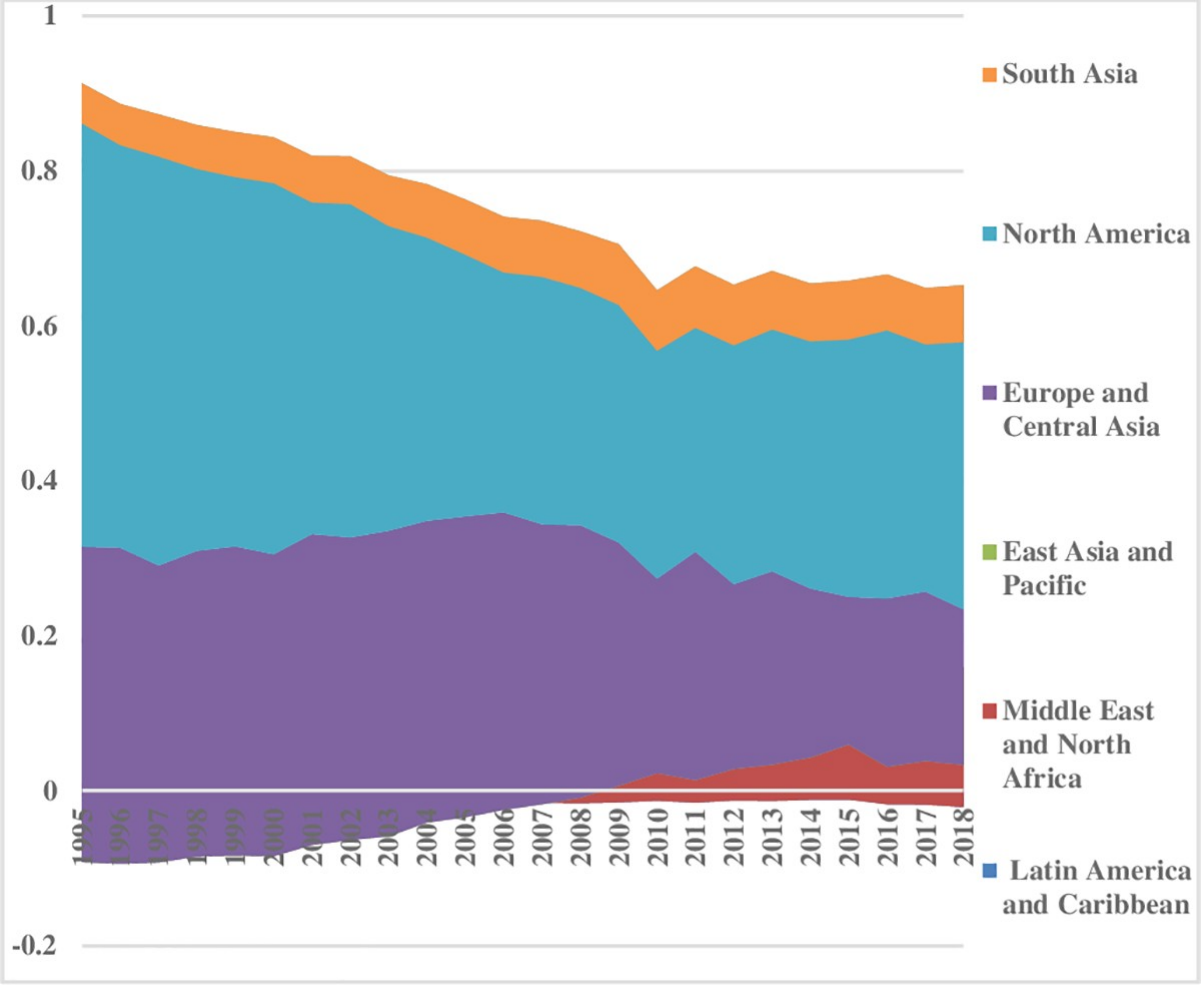
Pattern of inequality between the regions for natural gas.

**Fig 9:**
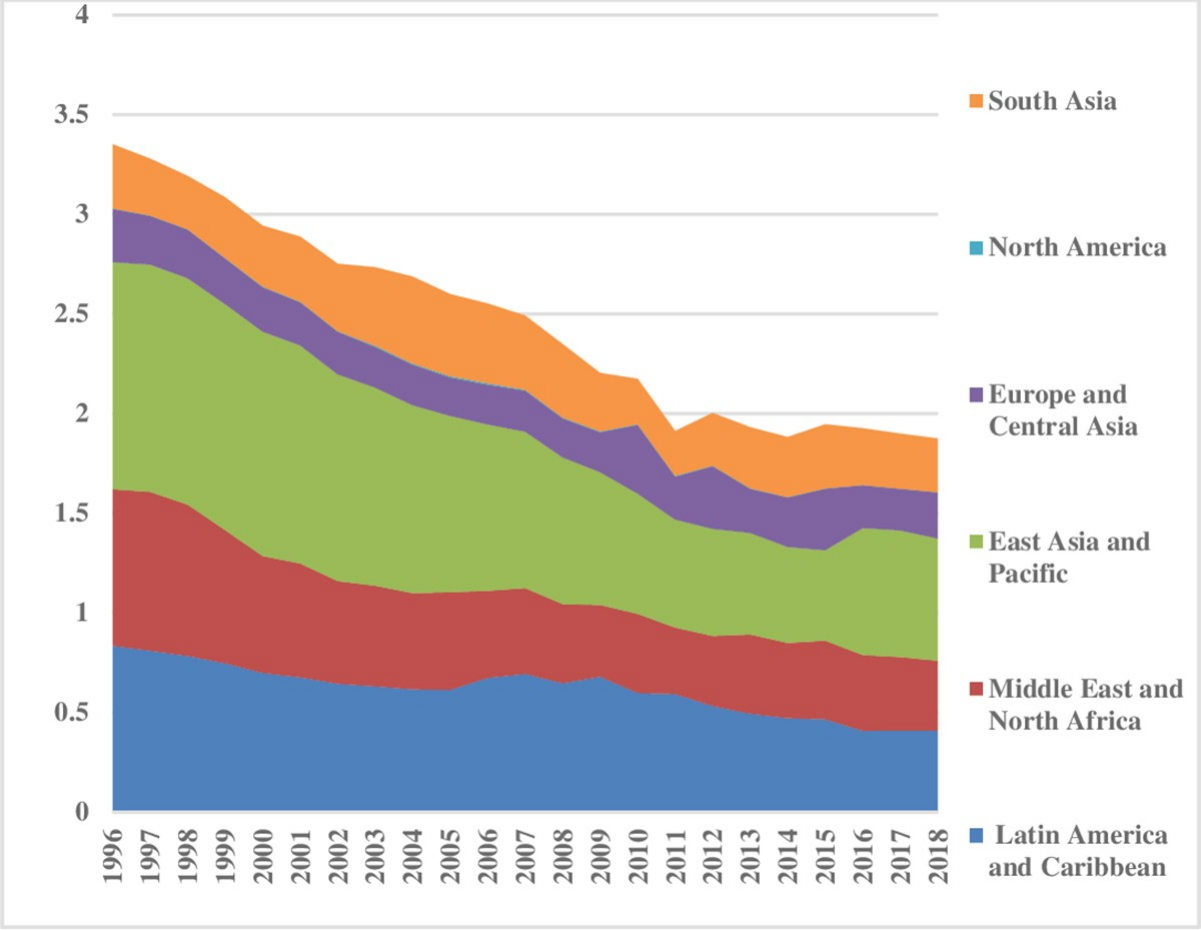
Pattern of inequality within the regions for natural gas.

**Fig 10:**
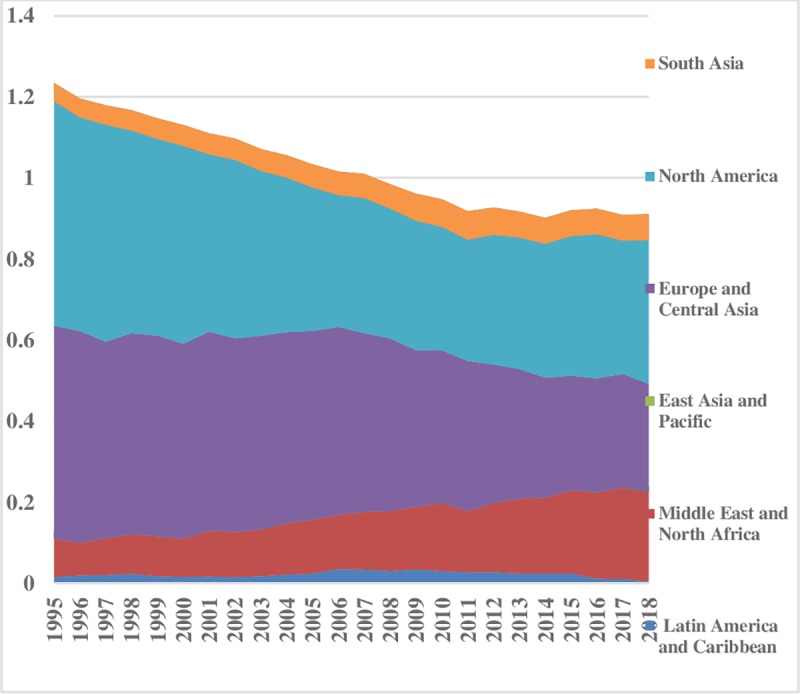
Pattern of total inequality for natural gas.

In the case of natural gas, we see that levels of consumption inequalities are decreasing in the world. However, as shown in Fig 8, there is the highest disparity between North America and Europe & Central Asian regions. But in other regions it is comparatively low. In contrast, a rising trend in Middle East & North Africa can be experienced over time. The inequality within the region is shown in Fig 9. East Asia & Pacific within the region has the highest inequalities in natural gas consumption, however, it has declined considerably over the sample period. Moreover, total energy inequality for natural gas consumption is presented in Fig 9. The lowest part of the index reveals minor total energy inequality for gas in Latin America & Caribbean, and East Asia & Pacific regions, while, South Asia is showing a steady trend in its total gas energy inequality. Total energy consumption is declining over time in sample regions (Fig 10).

**Fig 11:**
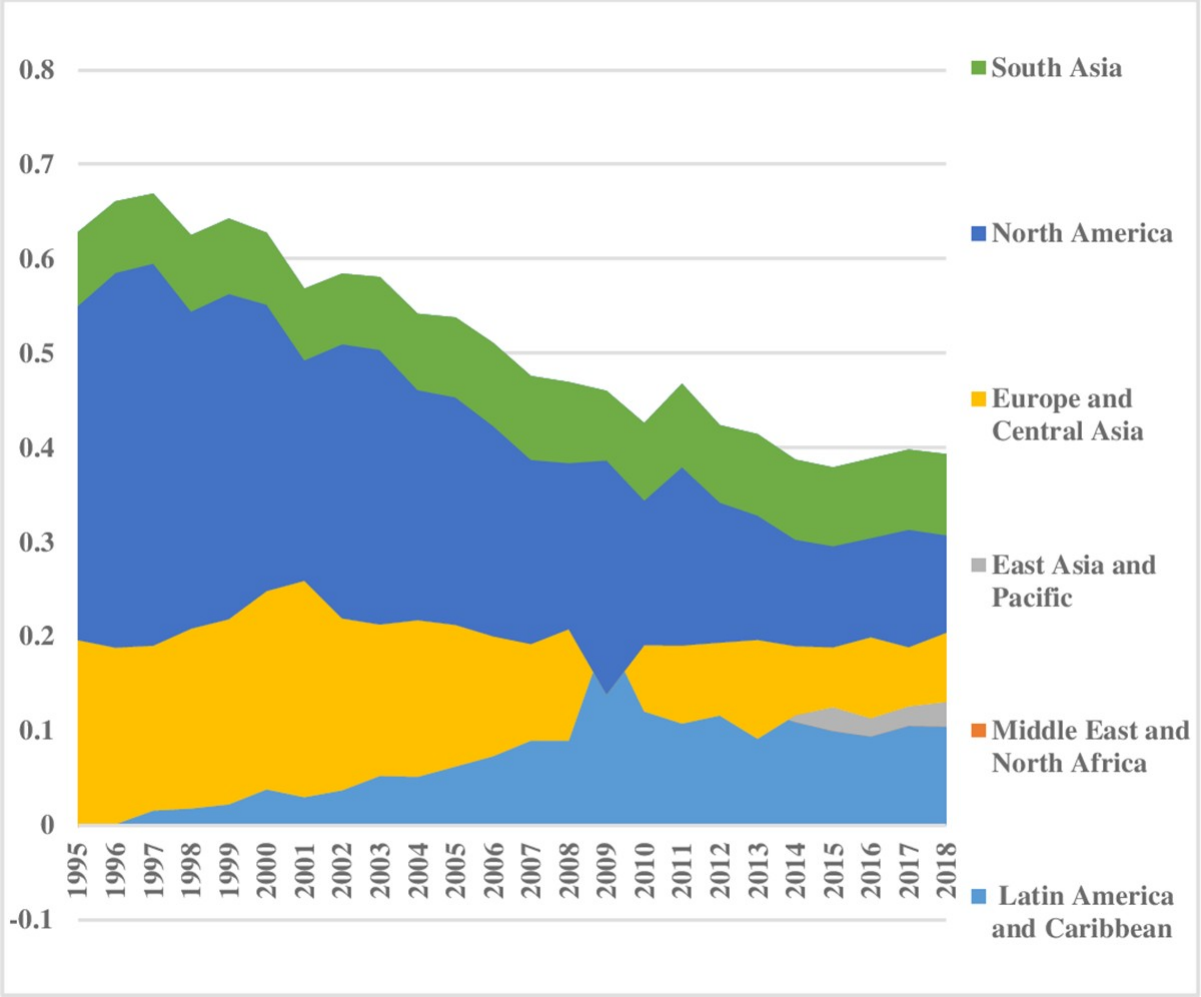
Pattern of inequality between the regions for hydroelectricity.

**Fig 12:**
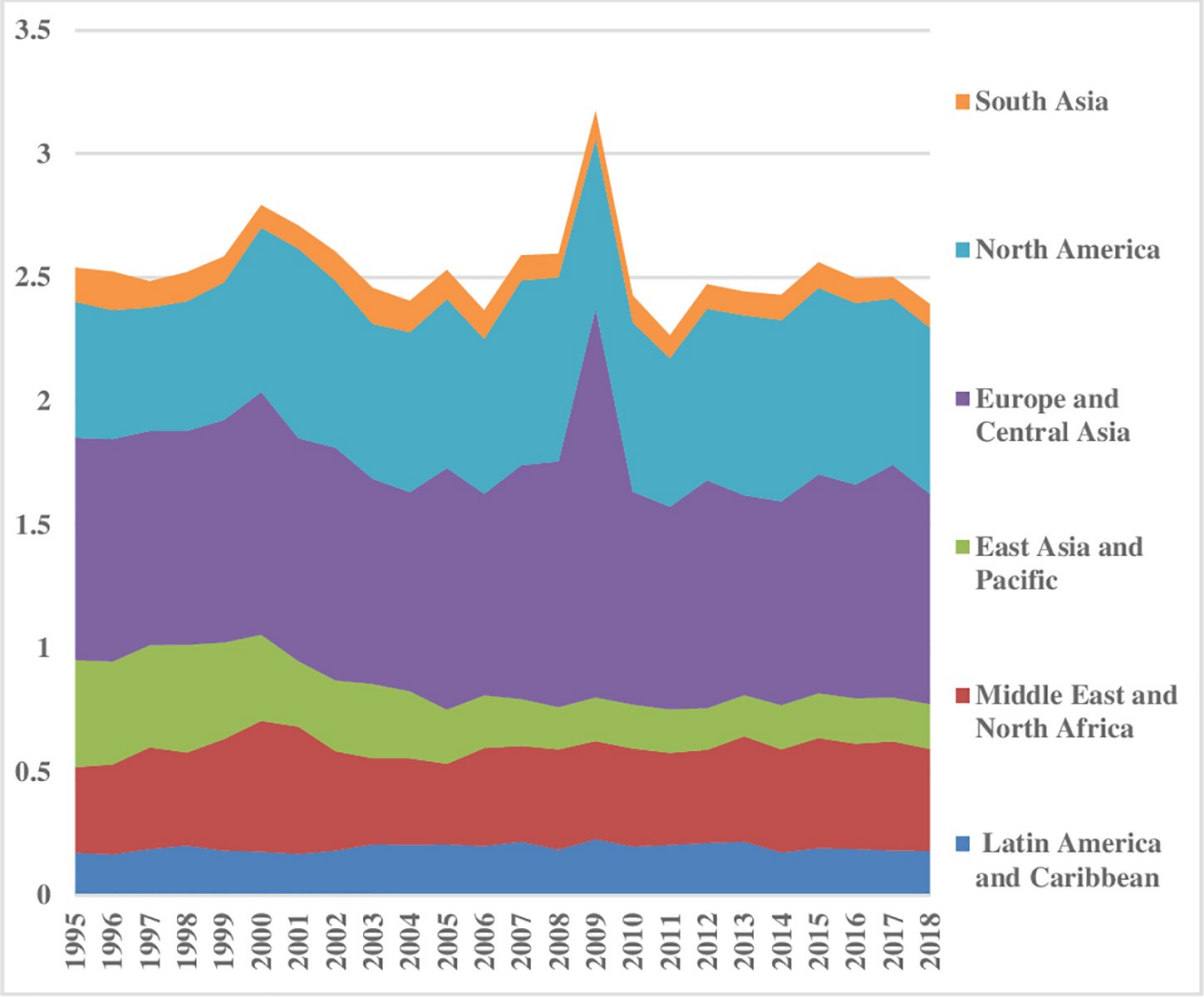
Pattern of inequality within the regions for hydroelectricity.

**Fig 13:**
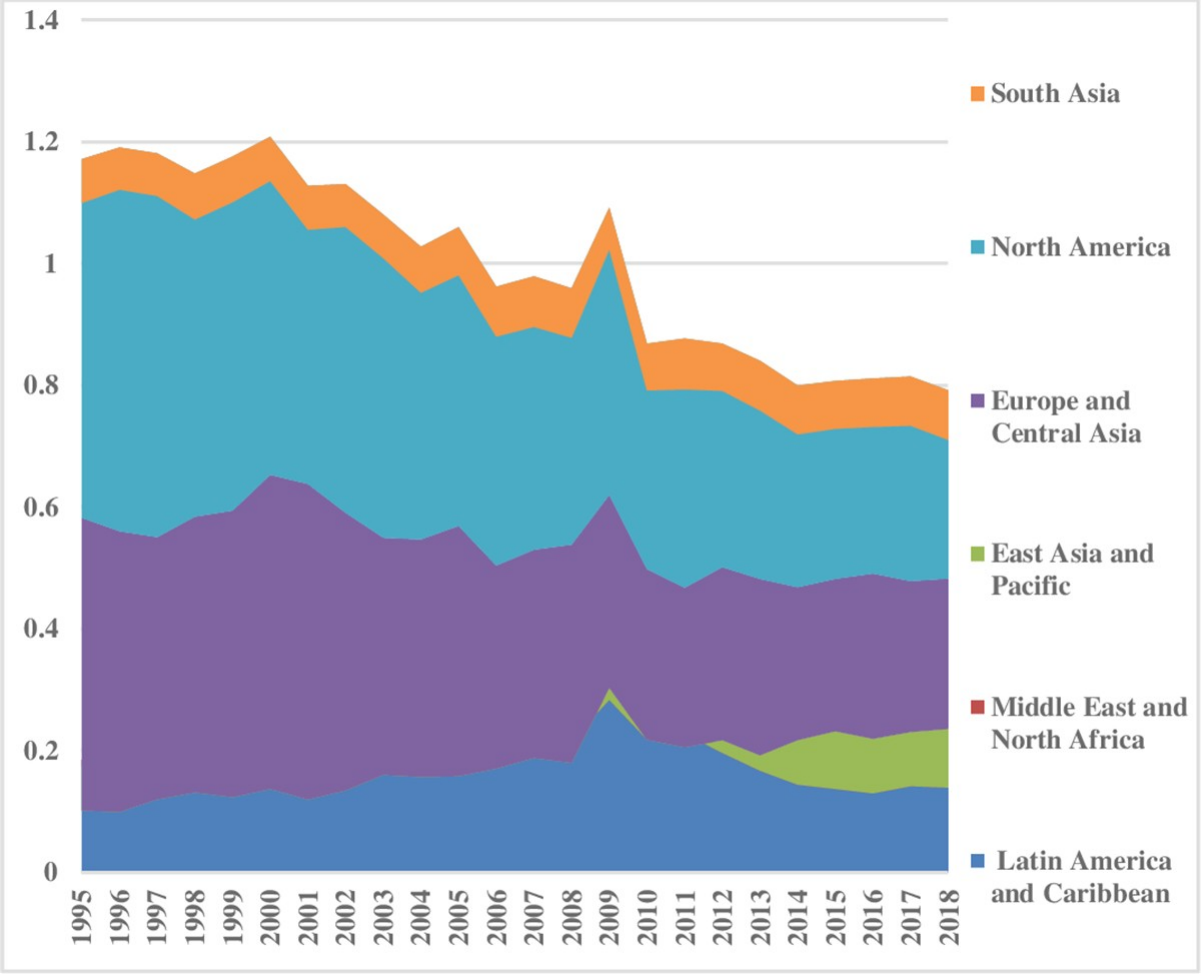
Pattern of total inequality for hydroelectricity.

With liberalization, inequality has fallen over the time in hydroelectricity consumption. The energy inequality between the regions for hydroelectricity is illustrated (Fig 11). The lower index value reveals less hydroelectricity consumption difference between the sampled regions and vice versa. The Middle East & North African region has the lowest inequality, while, East Asia & Pacific and South Asia have the normal level of energy inequality in hydroelectricity consumption between the regions. After 2011, South Asia follows an uprising trend between the regions, while, North America has the highest energy inequality in hydroelectricity consumption that has declined over the time. There is no big difference between Europe & Central Asia and Latin America & Caribbean regions energy consumption inequalities. Overall, hydroelectricity inequalities are decreasing over the time. Hydroelectricity inequality within the regions is illustrated in Fig 12. Latin America & Caribbean region is showing less energy consumption inequality within the regions that remained sustained over time. The hydroelectricity consumption inequality within East Asia & Pacific is low and persistent up to 2004; afterwards it followed a decreasing trend. There are not many disparities within South Asia. Compared to others, Europe & Central Asia and North America have significant inequalities in the consumption of hydroelectricity energy within the regions which remained consistent throughout the sample period. Total energy inequality in hydroelectricity consumption is depicted in Fig 13. The lower index value is indicating less inequality in the Middle East & North Africa, South Asia and East Asia & Pacific regions. Latin America & Caribbean region has normal total inequality in hydroelectricity consumption. North America and Europe & Central Asia portray a declining trend over the time in total energy inequality.

**Fig 14:**
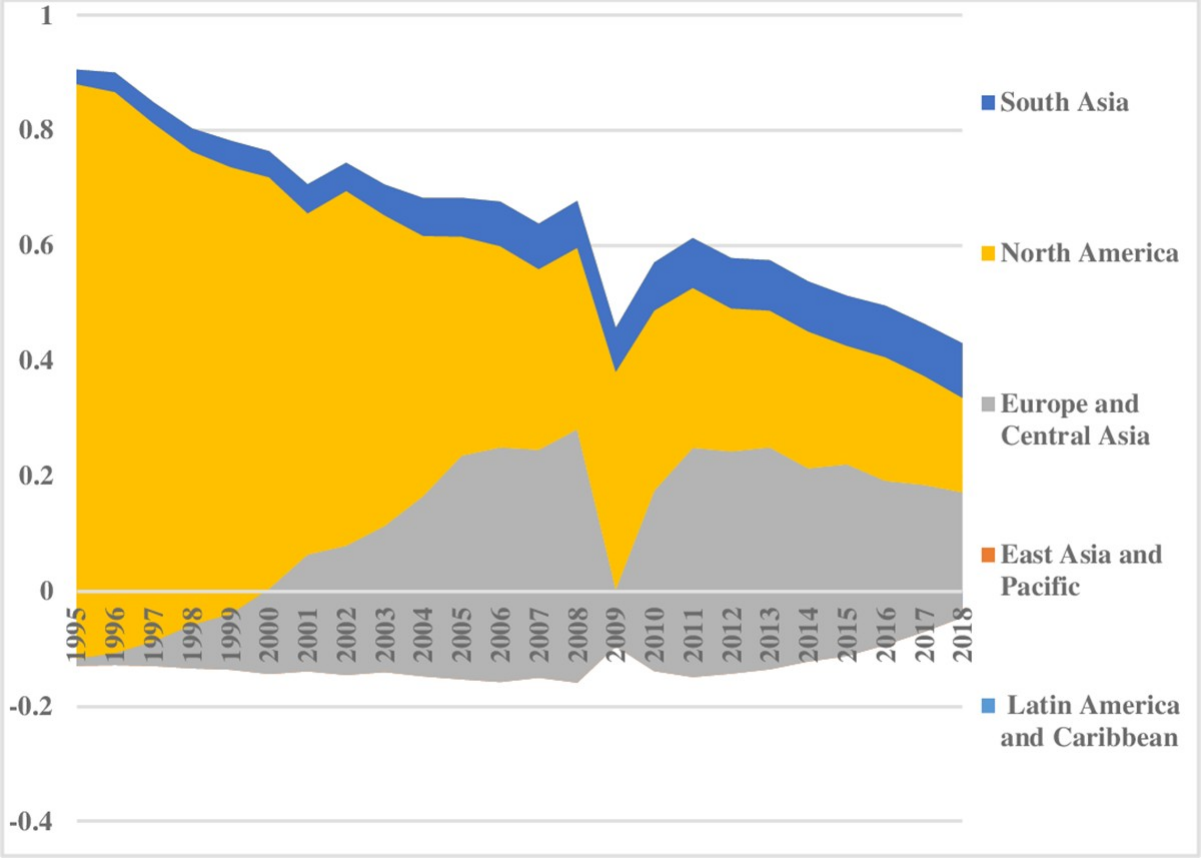
Pattern of inequality between the regions for renewable energy.

**Fig 15:**
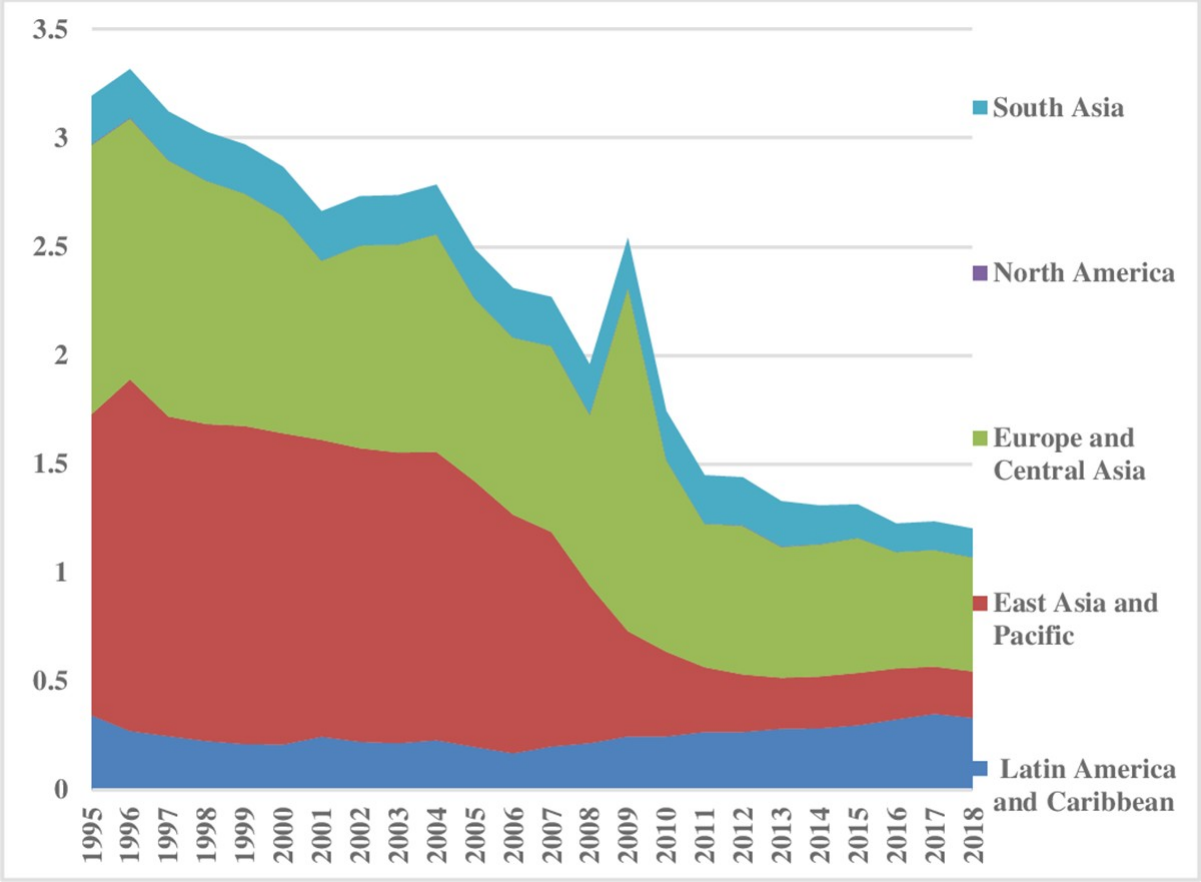
Pattern of inequality within the regions for renewable energy.

**Fig 16:**
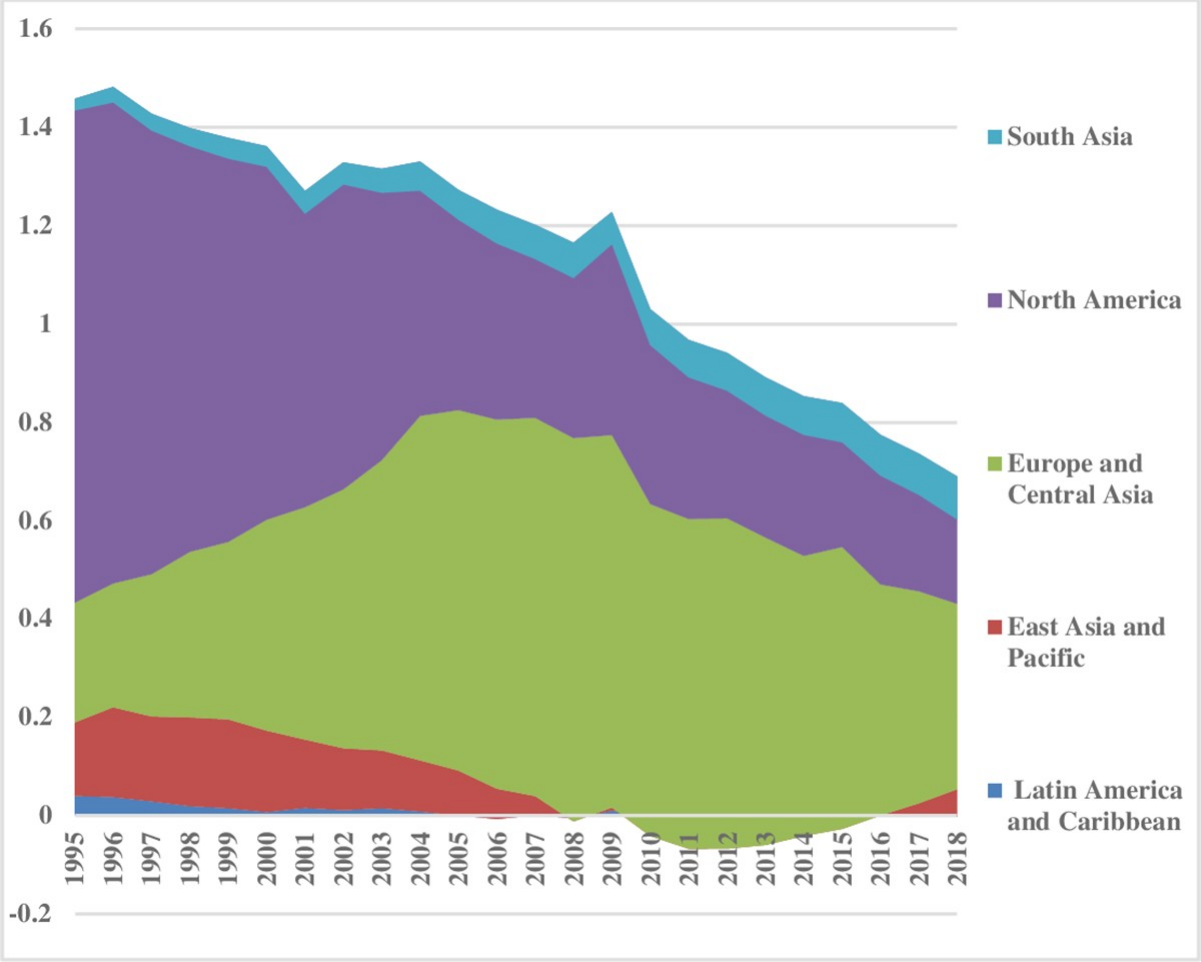
Pattern of total inequality for renewable.

It is a common belief that renewable energy is comparatively environment friendly. Over the time, the inequality in renewable energy consumption has decreased in the world. Renewable energy consumption inequality between the regions is demonstrated in Fig 14. Lower series of the index showed that there is a little discrepancy in Latin America & Caribbean relative to other regions. South Asia also has fewer disparities. North America has the highest inequality between the regions that has lowered over the time. Fig 15 outlined the inequality scenario for renewable energy consumption within regions. East Asia & Pacific has the highest disparities in renewable energy consumption within the region until 2004, afterward it has fallen over the time. A decreasing trend is visible in renewable consumption inequality within Europe & Central Asian region, while South Asia has a little discrepancy in the consumption of renewable energy, and its disparities have declined over the time, while disparities in Latin America have a tiny upward trend. Total energy inequality in renewable energy consumption is explained in Fig 16. The upper index value revealed that North America had the highest inequality in total renewable energy consumption, which is declining over the time while South Asia has minor inequality in total renewable consumption. However, the total renewable energy disparities in East Asia & Pacific have decreased over the time. Latin America & Caribbean has very minute inequality in total renewable energy consumption.

There is a clear decreasing trend in global inequalities in levels of energy consumption, especially within regions. It implies that the situation within regions is comparatively better than between world countries. But the inequality in energy access to resources did not vanish. According to the World Energy Council (2000) 40 percent of the world’s population has no systematic access to energy products in their homes [48]. Ultimately, unequal energy consumption patterns translate into different rates of greenhouse gas emissions throughout the world. People living in developing countries have unequal access to modern technology and clean energy resources [12, 14]. Energy is the biggest source of greenhouse gases emitted into the air that have widespread detrimental impacts on home-grown, regional and world eco-systems. Moreover, the evenhanded approach to address the problem of global climate change would be to define a standard per capita emissions rate and then put penalties on states that beat the standard [49].

## 4 Empirical assessments of energy inequalities on environmental quality

In order to conduct a comprehensive study, we have applied the system GMM to our study and also include the standard diagnostic tests mentioned above section. Different countries and regions are technologically at different stages but it data are not available for each country. Therefore, to minimize the technology differences, region level analysis will be more appropriate as these differences are comparatively low at regional level. It will also be important for robustness of the findings.

Region-wise environmental implications of between, within, and total energy consumption inequalities by oil are presented in Table 3. It is evident that countries are becoming more liberalized owing to trade operations and have access to various kinds of energy sources that lead to a decrease in inequality over time in oil, coal, natural gas, hydroelectricity, and renewable energy consumption. However, the energy industry still needs to be improved by giving global economies equal access to energy resources. Although we are observing a reduction in disparities in oil consumption, it is still insufficient to reduce environmental degradation. Oil energy inequality between the regions has a significant positive impact on carbon emissions in the world, Europe & Central Asia, North America (except within region), East Asia & Pacific, South Asia and Latin America & Caribbean regions. The energy inequality index also showed that North America has the greatest disparities between the regions. However, the Middle East & North Africa has a negative impact on the environmental quality. The majority of Middle Eastern countries, are endowed with natural resources and recognized as oil-producing and exporting countries. So possibly it has a modest impact of inequalities on the environment due to abundant resources.

**Table 3:** Results of oil energy inequality.

	**World**			**Europe and Central Asia**			**North America**			**Middle East and North Africa**		
**Models**	MD1	MD2	MD3	MD1	MD2	MD3	MD1	MD2	MD3	MD1	MD2	MD3
**C**	1.572***	4.487***	2.026***	-14.78***	-15.16***	-15.29***	-11.22	-10.93	-11.33	-12.43***	-11.9**	-12.26***
	(0.014)	(0.00)	(0.00)	(0.00)	(0.00)	(0.00)	(0.78)	(0.82)	(0.780)	(0.00)	(0.00)	(0.00)
**CO2**_**-1**_	.996***	.996***	.996***	.0458***	.052***	.0431***	.797***	.864***	.797***	.968***	.965***	.969***
	(0.00)	(0.00)	(0.00)	(0.000)	(0.000)	(0.00)	(0.00)	(0.00)	(0.00)	(0.000)	(0.000)	(0.00)
**GDP**	.00024***	-.000242***	-.0002***	.0003**	.0003	.0004***	.0002***	.00003***	.00002***	.0009***	.00010*	.00010***
	(0.00)	(0.00)	(0.00)	(0.027)	(0.01)	(0.00)	(0.00)	(0.00)	(0.00)	(0.00)	(0.00)	(0.00)
**GDPS**	-3.00^−07^***	-3.00^−07^***	-3.00^−07^***	-4.00^−07^***	-4.00^−07^***	-5.00^−07^***	-2.00^−07^***	-3.00^−07^***	-2.00^−06^***	-9.00^−07^***	-9.00^−07^***	-9.60^−07^***
	(0.00)	(0.00)	(0.00)	(0.01)	(0.025)	(0.01)	(0.00)	(0.00)	(0.00)	(0.00)	(0.00)	(0.00)
**BECS**	.8434***	----	----	2.488***	----	----	.889***	----	----	-1.26***	----	----
	(0.00)	----	----	(0.00)	----	----	(0.00)	----	----	(0.00)	----	----
**WECS**	----	.8629***	----	----	5.53	----	----	-5.46**	----	----	-.475***	----
	----	(0.00)	----	----	(0.00)	----	----	(0.031)	----	----	(0.00)	----
**TECS**	----	----	-.8145***	----	----	2.106***	----	----	.891***	----	----	-1.38***
	----	----	(0.00)	----	----	(0.003)	----	----	(0.00)	----	----	(0.00)
**TO**	1.085***	1.675***	1.169***	.5038**	.505**	.659***	.093***	.1063***	.093****	.056*	-.160***	.090***
	(0.00)	(0.00)	(0.00)	(0.03)	(0.03)	(0.00)	(0.00)	(0.00)	(0.00)	(0.08)	(0.00)	(0.003)
**PRG**	-1.843***	-1.821***	-1.829***	-.030	-.043	-.125	-1.527	-1.849	-1.49	-.268***	-.293***	-.274***
	(0.00)	(0.00)	(0.00)	(0.91)	(0.832)	(0.57)	(0.910)	(0.909)	(0.91)	(0.00)	(0.00)	(0.00)
**AR (1)**	-18.26	-18.26	-18.26	-0.96	-0.98	-0.96	-2.66	-2.52	-2.66	-2.90	-2.95	-2.95
	(0.000)	(0.000)	(0.000)	(0.336)	(0.329)	(0.336)	(0.008)	(0.012)	(0.008)	(0.004)	(0.003)	(0.003)
**AR (2)**	-1.44	1.51	-1.45	-1.38	-1.07	-1.36	-1.24	-1.66	-1.24	-1.87	-1.39	-1.80
	(0.150)	(0.131)	(0.146)	(0.166)	(0.287)	(0.174)	(0.215)	(0.096)	(0.215)	(0.061)	(0.165)	(0.072)
**Sargan test (p-value)**	(0.873)	(0.862)	(0.869)	(0.537)	(0.492)	(0.597)	(0.228)	(0.178)	(0.228)	(0.431)	(0.431)	(0.412)
**No. Obs**	1311	1311	1311	598	598	598	46	46	46	184	184	184
	**East Asia and pacific**				**South Asia**				**Latin America and Caribbean**			
**Models**	**MD1**	**MD2**		**MD3**	**MD1**	**MD2**		**MD3**	**MD1**	**MD2**		**MD3**
**C**	-16.566***	-16.22***		-17.87***	.432*	-1.498***		.428*	1.248***	1.032***		-.527***
	(0.00)	(0.00)		(0.00)	(0.058)	(0.00)		(0.052)	(0.00)	(0.00)		(0.00)
**CO2**_**-1**_	.904***	.996***		.993***	.993***	1.082***		1.008***	.945***	.946***		.946***
	(0.00)	(0.00)		(0.00)	(0.00)	(0.00)		(0.00)	(0.00)	(0.00)		(0.00)
**GDP**	.00016**	.00015***		.00014***	.00060***	.0052***		.0059***	.00037***	.00036***		.00016***
	(0.00)	(0.00)		(0.00)	(0.00)	(0.00)		(0.00)	(0.00)	(0.00)		(0.00)
**GDPS**	-.00000021***	-.0000002***		-.00000018***	-.00000015***	-.0000002***		-.00000015***	-.323***	-.284***		-.166***
	(0.00)	(0.00)		(0.00)	(0.00)	(0.00)		(0.00)	(0.00)	(0.00)		(0.00)
**BECS**	6.756***	----		----	4.25***	----		----	-8.230***	----		----
	(0.00)	----		----	(0.00)	----		----	(0.08)	----		----
**WECS**	----	.933***		----	----	2.507****		----	----	8.579***		----
	----	(0.00)		----	----	(0.00)		----	----	(0.00)		----
**TECS**	----	----		1.22	----	----		4.39***	----	----		10.58***
	----	----		(0.171)	----	----		(0.00)	----	----		(0.00)
**TO**	.809***	.663***		.936***	.455***	.948***		.477***	1.457***	1.499***		1.545***
	(0.00)	(0.00)		(0.00)	(0.00)	(0.00)		(0.00)	(0.00)	(0.00)		(0.00)
**PRG**	-.0168	-.057***		-.0280***	-.053**	-.0391*		-.031	-.506***	-.517***		-.303***
	(0.334)	(0.00)		(0.000)	(0.032)	(0.084)		(0.146)	(0.00)	(0.00)		(0.00)
**AR (1)**	-1.20	-1.55		-8.99	-2.79	-3.00		-2.78	-5.97	-6.00		-5.91
	(0.231)	(0.121)		(0.000)	(0.005)	(0.003)		(0.005)	(0.000)	(0.000)		(0.000)
**AR (2)**	-1.44	-0.59		-1.23	-0.90	-1.10		-0.86	0.85	0.86		0.85
	(0.150)	(0.554)		(0.219)	(0.370)	(0.273)		(0.387)	(0.393)	(0.389)		(0.395)
**Sargan test (p-value)**	(0.339)	(0.318)		(0.295)	(0.642)	(0.714)		(0.630)	(0.818)	(0.823)		(0.822)
**No. Obs**	230	230		230	69	69		69	184	184		184

Prob-values are stated in parenthesis, ***,**,* show the significance level at 1%,5% and 10% respectively. C = constant, MD1 = between energy consumption inequality (BECS). MD2 = energy consumption inequality within regions (WECS), MD3 = total energy consumption inequality (TECS), GDP, gross domestic income, GDPS = Square of gross domestic income (GDP and GDPS for EKC (Environmental Kuznets Curve), TO = trade openness, PRG = political regimes.

The inequality index also showed less severity in its consumption which has a declining trend after 2015. The energy inequality of oil within region also decreases the environmental quality. North America and Middle East & North Africa inequalities within regions have no detrimental impact on the environment. As inequality index of oil consumption also showed fewer disparities within the North America region. Total oil consumption inequality is also increasing environmental pollution in the world and all regions except the Middle East & North Africa. These results depict that inequalities in oil consumption accrue sever damaging impact on environmental quality as indicated by (Shafiei et al; Tang et al)[50, 51] which also showed that Non-renewable and total energy consumption is decreasing environment quality. Trade increases the environmental degradation in the world, Europe & Central Asia, South Asia, North America, East Asia & Pacific, Latin America & Caribbean regions except within the Middle East & North Africa region. We have also captured the dynamism of the Environmental Kuznets Curve as it plights the linkage between carbon emission and economic growth. Results reported in Table 3 show that environmental degradation increases at the initial stage of the development. These results are consistent with [22]. However, in the latter phase of development environmental degradation improves, as the impact of GDPS is negative in all sample regions. However, the impact of political regimes is statistically negative in the world and across all regions. It implies that countries having more liberal democracy take action to reduce carbon emissions.

Coal is the largest source of energy; however, most of the countries are striving to switch from coal to cleaner sources of energy along with the demand for higher economic growth, especially in developing countries. The inequality impact of coal consumption is reported in Table 4. The results show that coal consumption inequality also degrades the environment in the world, North America, South Asia, East Asia & Pacific, Latin America & Caribbean, and Europe & Central Asia. However, the world sample does not show a significant inequality impact of coal consumption in the case of within regions. However, in the case of within the North American region, we find a negative impact of energy inequality on the environment. The inequality index within region also indicates that North America has fewer disparities in coal consumption. The total energy disparities of coal consumption have a perilous impact on the environment in the case of the world and all six regions as the coefficients’ sign are positive. Moreover, the Middle East & North Africa does not show significant inequality impact of coal consumption on the environment. Trade also does not improve the environmental quality in the case of World and East Asia & Pacific, South Asia, and Latin America & Caribbean, Europe & Central Asia and North America. However, political regimes are again a beneficial indicator to control the pollution as it has a negative impact on carbon emission. In coal source, the outcomes reported in Table 4 sustain the cogency of the EKC hypothesis among the selected sample countries of the world and across six regions.

**Table 4:** Results of coal energy inequality.

	**World**			**Europe and Central Asia**			**North America**			**Middle East and North Africa**		
**Models**	**MD1**	**MD2**	**MD3**	**MD1**	**MD2**	**MD3**	**MD1**	**MD2**	**MD3**	**MD1**	**MD2**	**MD3**
**C**	2.048***	-4.403	-.513	-15.53***	-16.50***	-16.74***	-11.029	-10.91	-11.25	-11.84***	-12.03***	-11.03***
	(0.00)	(0.00)	(0.35)	(0.00)	(0.00)	(0.00)	(0.81)	(0.81)	(0.79)	(0.00)	(0.00)	(0.00)
**CO2**_**-1**_	.996***	.996***	.996***	.634***	.645***	.635***	.829***	.863***	.836***	.970***	.964***	.966***
	(0.00)	(0.00)	(0.00)	(0.00)	(0.00)	(0.00)	(0.00)	(0.00)	(0.00)	(0.00)	(0.00)	(0.00)
**GDP**	-.00024***	-.00014***	-.00019***	.00033***	.0004***	.00048***	.0002***	.00023***	.00029***	.00087***	.00074***	.00082***
	(0.00)	(0.00)	(0.00)	(0.00)	(0.00)	(0.00)	(0.00)	(0.00)	(0.00)	(0.00)	(0.00)	
**GDPS**	-3.0^−07^***	-1.3^−07^***	-2.2^−07^***	-5.0^−07^***	-6.1^−07^***	-6.1^−07^***	-2.3^−07^***	-2.0^−07^***	-3.0^−07^***	-7.3^−07^***	-5.2^−07^***	-6.6^−07^***
	(0.00)	(0.00)	(0.00)	(0.01)	(0.00)	(0.00)	(0.00)	(0.00)	(0.00)	(0.00)	(0.00)	(0.00)
**BECS**	1.429***	----	----	1.05***	----	----	4.43	----	----	6.217	----	----
	(0.00)	----	----	(0.00)	----	----	(0.39)	----	----	(0.13)	----	----
**WECS**	----	-.0377	----	----	.209***	----	----	-.689	----	----	.136	----
	----	(0.17)	----	----	(0.00)	----	----	(0.00)	----	----	(0.428)	----
**TECS**	----	----	.734***	----	----	.390	----	----	16.97***	----	----	8.76
	----	----	(0.00)	----	----	(0.421)	----	----	(0.00)	----	----	(0.116)
**TO**	1.19***	.4182***	.852***	.663***	.748**	.824***	.110***	.105	.111	.145***	-.116***	.138***
	(0.00)	(0.00)	(0.00)	(0.00)	(0.00)	(0.00)	(0.00)	(0.00)	(0.00)	(0.00)	(0.00)	(0.00)
**PRG**	-1.804***	-.622***	-1.434***	-.142	-.432***	-.337*	-1.99	-1.46	-2.00	-.375***	-.374***	-.395***
	(0.00)	(0.00)	(0.00)	(0.40)	(0.00)	(0.09)	(0.89)	(0.92)	(0.88)	(0.00)	(0.00)	(0.00)
**AR (1)**	-18.26	-18.26	-18.26	-6.77	-6.96	-6.70	-1.20	-3.99	-1.24	-3.02	-2.89	-2.96
	(0.000)	(0.000)	(0.000)	(0.000)	(0.000)	(0.000)	(0.229)	(0.000)	(0.215)	(0.003)	(0.004)	(0.003)
**AR (2)**	-1.40	1.51	1.51	0.56	0.55	0.56	-0.04	-0.07	0.05	-1.60	-1.46	-1.23
	(0.162)	(0.131)	(0.131)	(0.575)	(0.582)	(0.575)	(0.967)	(0.942)	(0.959)	(0.110)	(0.143)	(0.218
**Sargan test (p-value)**	(0.873)	(0.862)	(0.869)	(0.316)	(0.420)	(0.461)	(0.407)	(0.492)	(0.504)	(0.442)	(0.412)	(0.433)
**No. Obs**	1311	1311	1311	598	598	598	46	46	46	184	184	184
**East Asia and pacific**					**South Asia**				**Latin America and Caribbean**			
**Models**	**MD1**	**MD2**	**MD3**		**MD1**	**MD2**		**MD3**	**MD1**	**MD2**		**MD3**
**C**	-17.48***	-16.20***	-17.51***		1.247***	.394		.152	-.070	.622**		.974***
	(0.00)	(0.00)	(0.00)		(0.00)	(0.20)		(0.72)	(0.83)	(0.01)		(0.00)
**CO2**_**-1**_	.998***	.994***	.998***		.964***	.966***		.972***	.945***	.950***		.944***
	(0.000)	(0.000)	(0.00)		(0.00)	(0.00)		(0.00)	(0.00)	(0.00)		(0.00)
**GDP**	.00014***	.00015***	.00014***		.00053***	.0054***		.0052***	.00015***	.00014***		.00014***
	(0.00)	(0.00)	(0.00)		(0.00)	(0.00)		(0.00)	(0.00)	(0.00)		(0.00)
**GDPS**	-.00000019***	-.00000019***	-.00000018***		-.00000014***	-.00000014***		-.00000014***	-.00000013***	-.00000011***		-.00000012***
	(0.00)	(0.00)	(0.00)		(0.00)	(0.00)		(0.00)	(0.00)	(0.00)		(0.00)
**BECS**	.991***	----	----		6.34***	----		----	35.80	----		----
	(0.00)	----	----		(0.00)	----		----	(0.00)	----		----
**WECS**	----	6.637***	----		----	4.383***		----	----	1.580***		----
	----	(0.00)	----		----	(0.00)		----	----	(0.00)		----
**TECS**	----	----	1.023***		----	----		3.19**	----	----		20.70**
	----	----	(0.00)		----	----		(0.03)	----	----		(0.01)
**TO**	.825***	.880***	.813		1.058***	1.01***		.973***	.405***	.291***		.222***
	(0.00)	(0.00)	(0.00)		(0.00)	(0.00)		(0.00)	(0.00)	(0.00)		(0.00)
**PRG**	-.0086	-.064**	-.015		-.0305	-.0261		-.034	-.117***	-.110***		-.117***
	(0.67)	(0.01)	(0.46)		(0.20)	(0.35)		(0.12)	(0.00)	(0.00)		(0.00)
**AR (1)**	-1.74	-1.56	-1.74		-3.46	-3.99		-3.26	-6.18	-6.02		-6.23
	(0.083)	(0.118)	(0.083)		(0.001)	(0.000)		(0.001)	(0.000)	(0.000)		(0.000)
**AR (2)**	0.05	-1.71	0.25		-1.48	-1.01		-1.30	0.86	0.89		0.78
	(0.956)	(0.087)	(0.805)		(0.139)	(0.312)		(0.192)	(0.389)	(0.376)		(0.435)
**Sargan test (p-value)**	(0.786)	(0.762)	(0.783)		(0.417)	(0.537)		(0.404)	(0.812)	(0.819)		(0.748)
**No. Obs**	240	240	240		69	69		69	184	184		184

See note under Table 3.

Natural gas is another important energy source and has major environmental implications. The results of gas consumption inequalities are described in Table 5. Inequality in natural gas consumption is also not environment friendly as the coefficient of between regions inequality (BECS) is positive in the world, Europe & Central Asia, North America, Middle East & North Africa, East Asia & Pacific, South Asia, and Latin America & Caribbean regions. However, within regions inequalities of gas consumption have a negative influence on environmental quality in the case of Europe & Central Asia and East Asia & Pacific. Inequality index values also endorse that Europe & Central Asia has fewer disparities within regions in natural gas consumption. In contrast, within regions inequalities (WECS) have a positive impact on the world, North America, Middle East & North Africa, South Asia and Latin America & the Caribbean regions’ environment. Moreover, total energy inequality also positively contributes to increasing the pollution in the case of the world, Europe & Central Asia, Middle East & North Africa, North America, South Asia and Latin America & Caribbean regions. Once again, trade has no role in improving the environment. The political regimes show power to control environmental degradation. The results of GDP and square of GDP reported in Table 5 again validate the inverted U-Shaped EKC curve.

**Table 5:** Results of gas energy inequality.

	**World**			**Europe and Central Asia**			**North America**			**Middle east and north Africa**		
**Models**	**MD1**	**MD2**	**MD3**	**MD1**	**MD2**	**MD3**	**MD1**	**MD2**	**MD3**	**MD1**	**MD2**	**MD3**
**C**	3.197***	.843***	-2.288***	-15.70***	-16.3***	-14.69***	-10.971	-10.820	-11.01	-12.04***	-11.817***	-12.03***
	(0.00)	(0.00)	(0.00)	(0.00)	(0.00)	(0.00)	(0.86)	(0.86)	(0.86)	(0.00)	(0.00)	(0.00)
**CO2**_**-1**_	.985***	.993***	.993***	.632***	.631***	.633***	.875***	.856***	.875***	.969***	.970***	.969***
	(0.00)	(0.00)	(0.00)	(0.00)	(0.00)	(0.00)	(0.00)	(0.00)	(0.00)	(0.00)	(0.00)	(0.00)
**GDP**	-.00024***	-.00019***	-.00014***	.0004***	.0005***	.0003***	.0003***	.00015***	.00028***	.0008***	.00077***	.00052***
	(0.00)	(0.00)	(0.00)	(0.000)		(0.00)		(0.00)	(0.000)	(0.00)	(0.00)	(0.00)
**GDPS**	-3.0^−07^***	-2.1^−07^***	-1.3^−07^***	-6.2^−07^***	-6.1^−07^***	-6.0^−07^***	-3.2^−07^***	-1.8^−07^***	-3.9^−07^***	-5.4^−07^***	-5.8^−07^***	-2.4^−07^***
	(0.00)	(0.00)	(0.00)	(0.00)	(0.00)	(0.00)	(0.00)	(0.00)	(0.000)	(0.00)	(0.00)	(0.00)
**BECS**	1.096***	----	----	.966***	----	----	.1769	----	----	.584***	----	----
	(0.00)	----	----	(0.00)	----	----	(0.374)	----	----	(0.00)	----	----
**WECS**	----	.2124***	----	----	-.878**	----	----	19.01***	----	----	.190***	----
	----	(0.00)	----	----	(0.028)	----	----	(0.00)	----	----	(0.00)	----
**TECS**	----	----	.899***	----	----	1.076***	----	----	.191***	----	----	.874***
	----	----	(0.00)	----	----	(0.00)	----	----	0.338	----	----	(0.00)
**TO**	1.403***	1.15***	.737***	.716***	.752***	.560***	.089***	.083***	.089***	.124***	-.139***	.109***
	(0.00)	(0.00)	(0.00)	(0.00)	(0.00)	(0.019)	(0.00)	(0.00)	(0.00)	(0.00)	(0.00)	(0.00)
**PRG**	-1.814***	-1.432***	-.642***	-.065	-.38	-.24	-1.934	-1.025	-1.932	-.386***	-.403***	-.387***
	(0.00)	(0.00)	(0.00)	(0.77)	(0.028)	(0.291)	(0.930)	(0.963)	0.930	(0.00)	(0.00)	(0.00)
**AR (1)**	-16.84	-24.91	-23.61	-6.66	-6.81	-6.65	-0.95	-3.41	-0.99	-2.93	-3.04	-2.91
	(0.000)	(0.000)	(0.000)	(0.000)	(0.000)	(0.000)	(0.044)	(0.001)	(0.022)	(0.003)	(0.002)	(0.004)
**AR (2)**	-1.60	-1.13	1.41	0.56	0.60	0.56	-0.42	-0.30	-0.42	-1.15	-1.08	-0.67
	(0.110)	(0.258)	(0.160)	(0.573)	(0.548)	(0.577)	(0.673)	(0.764)	(0.677)	(0.250)	(0.280)	(0.505)
**Sargan test (p-value)**	(0.965)	(0.438)	(0.439)	(0.688)	(0.674)	(0.690)	(0.608)	(0.625)	(0.609)	(0.415)	(0.405)	(0.412)
**No. Obs**	1311	1311	1368	598	598	598	46	46	46	184	184	184
	**East Asia and Pacific**				**South Asia**				**Latin America and Caribbean**			
**Models**	**MD1**	**MD2**		**MD3**	**MD1**	**MD2**		**MD3**	**MD1**	**MD2**		**MD3**
**C**	-16.31***	-16.37***		-16.70***	-2.753***	-3.48***		-.328	.8015***	1.788***		.723***
	(0.00)	(0.00)		(0.00)	(0.00)	(0.00)		(0.19)	(0.00)	(0.00)		(0.00)
**CO2**_**-1**_	.992***	.996***		.992***	.969***	.967***		.962***	.947***	.947***		.955***
	(0.00)	(0.00)		(0.00)	(0.00)	(0.00)		(0.00)	(0.00)	(0.00)		(0.00)
**GDP**	.00014***	.00015***		.00014***	.00055***	.00059***		.00052**	.0001***	.00012***		.00013***
	(0.00)	(0.00)		(0.00)	(0.00)	(0.00)		(0.00)	(0.00)	(0.00)		(0.00)
**GDPS**	-.00000018***	-.00000021***		-.00000018***	-.00000015***	-.0000002***		-.0000001***	-.00000011***	-.000001***		-.0000001***
	(0.00)	(0.00)		(0.00)	(0.00)	(0.00)		(0.00)	(0.00)	(0.00)		(0.00)
**BECS**	18.83**	----		----	33.67***	----		----	2.77	----		----
	(0.00)	----		----	(0.00)	----		----	(0.143)	----		----
**WECS**	----	-1.141***		----	----	2.39***		----	----	.470***		----
	----	(0.00)		----	----	(0.00)		----	----	(0.00)		----
**TECS**	----	----		1.42**	----	----		10.166***	----	----		8.94***
	----	----		(0.01)	----	----		(0.00)	----	----		(0.00)
**TO**	.644***	.778***		.629***	.570***	.708***		1.100***	.183***	.063		.239***
	(0.00)	(0.00)		(0.00)	(0.00)	(0.00)		(0.00)	(0.00)	(0.28)		(0.00)
**PRG**	-.1093***	.025***		-.197***	.0122	-.0176		.0339	-.168***	-.186***		-.138***
	(0.00)	(0.18)		(0.00)	(0.53)	(0.45)		(0.13)	(0.00)	(0.00)		(0.00)
**AR (1)**	-1.50	-1.60		-1.91	-5.02	-3.65		-5.09	-5.74	-5.67		-6.11
	(0.133)	(0.110)		(0.056)	(0.000)	(0.000)		(0.00)	(0.000)	(0.000)		(0.000)
**AR (2)**	0.87	0.90		-1.21	-1.08	-1.54		-0.89	-0.46	-0.65		-1.12
	(0.385)	(0.369)		(0.226)	(0.280)	(0.123)		(0.372)	(0.646)	(0.517)		(0.264)
**Sargan test (p-value)**	(0.732)	(0.783)		(0.733)	(0.480)	(0.605)		(0.546)	(0.827)	(0.845)		(0.910)
**No. Obs**	230	240		240	69	69		69	184	184		184

See note under Table 3.

Hydroelectricity is a competitive source of renewable energy. In the coming years, its consumption is anticipated to rise as a cleaner source. However, its consumption inequality fails to yield better environmental outcomes. The results of energy consumption inequalities by hydroelectricity sources are presented in Table 6. The results show that the inequality between regions is harmful to the environment in the case of all regions except the world and the Middle East & North Africa. However, the inequality of hydroelectricity consumption within region is negative in the world, Middle East & North Africa, East Asia & Pacific. It is evident from the hydroelectricity inequality index that East Asia & pacific has minor disparities within region which are decreasing over the time. Possibly, due to the large production of China in hydroelectric power the inequality within East Asia & pacific regions is on decreasing path. The utilization of hydroelectricity in Asian countries is increasing that emit less emission [52, 53]. Moreover, total energy inequality also increases environmental degradation positively in all regions except the world and Middle East & North Africa. In the case of hydroelectricity energy source, the coefficient of trade remained almost positive except the Middle East and North Africa, North America and to some extent in the world. However, the impact of political regimes on the environment is negative. Moreover, the results of GDP and square of GDP indorsed our previous results and confirm the inverted U-Shaped EKC curve in the sampled countries.

**Table 6:** Results of hydroelectricity energy inequality.

	**World**			**Europe and Central Asia**			**North America**			**Middle East and North Africa**		
**Models**	**MD1**	**MD2**	**MD3**	**MD1**	**MD2**	**MD3**	**MD1**	**MD2**	**MD3**	**MD1**	**MD2**	**MD3**
**C**	2.508***	-3.249***	-2.914***	-15.07***	-15.3***	-16.12***	-10.55	-10.87	-10.125	-12.08***	-11.86***	-12.25***
	(0.00)	(0.00)	(0.00)	(0.00)	(0.00)	(0.00)	(0.89)	(0.87)	(0.88)	(0.00)	(0.00)	(0.00)
**CO2**_**-1**_	.986***	.986***	.986***	.635***	.648***	.633***	.863***	.879***	.849***	.969***	.967***	.967***
	(0.00)	(0.00)	(0.00)	(0.00)	(0.00)	(0.00)	(0.00)	(0.00)	(0.00)	(0.00)	(0.00)	(0.00)
**GDP**	-.00024***	-.00014***	-.00014***	.00032***	.00034**	.00045***	.00022***	.00025***	.00017***	.00083***	.00088***	.00010***
	(0.00)	(0.00)	(0.00)	(0.01)	(0.016)	(0.00)	(0.00)	(0.00)	(0.00)	(0.00)	(0.00)	(0.00)
**GDPS**	-3.0^−07^***	-1.3^−07^***	-1.3^−07^***	-4.0^−07^***	-4.0^−07^***	-6.0^−07^***	-2.3^−07^***	-3.1^−07^***	-1.8^−07^***	-6.7^−07^***	-7.4^−07^***	-8.9^−07^***
	(0.00)	(0.00)	(0.00)	(0.02)	(0.012)	(0.00)	(0.00)	(0.00)	(0.00)	(0.00)	(0.00)	(0.00)
**BECS**	-.864***	----	----	.747***	----	----	1.159***	----	----	-8.84	----	----
	(0.00)	----	----	(0.00)	----	----	(0.00)	----	----	(0.139)	----	----
**WECS**	----	-.284***	----	----	.140	----	----	.128	----	----	-.278**	----
	----	(0.00)	----	----	(0.183)	----	----	(0.362)	----	----	(0.01)	----
**TECS**	----	----	-.5621***	----	----	.477***	----	----	1.135***	----	----	-8.33
	----	----	(0.00)	----	----	(0.00)	----	----	(0.00)	----	----	(0.257)
**TO**	1.331***	-.562***	-.689***	.549**	.572**	.731***	-.060***	-.0941***	-.0546***	-.142***	-.150***	-.169***
	(0.00)	(0.00)	(0.00)	(0.02)	(0.01)	(0.00)	(0.00)	(0.00)	(0.00)	(0.00)	(0.00)	(0.00)
**PRG**	-1.808***	-.599***	-.602***	-.142	-.348**	-.176	-1.909	-1.742	-1.797	-.395**	-.377**	-.213***
	(0.00)	(0.00)	(0.00)	(0.39)	(0.02)	(0.40)	(0.94)	(0.93)	(0.94)	(0.00)	(0.00)	(0.00)
**AR (1)**	-14.89	-14.86	-14.81	-6.84	-7.05	-6.67	-0.33	-0.29	-0.34	-2.93	-2.98	-3.21
	(0.000)	(0.000)	(0.000)	(0.000)	(0.000)	(0.000)	(0.743)	(0.776)	(0.737)	(0.003)	(0.003)	(0.001)
**AR (2)**	-1.56	1.56	1.59	0.28	0.26	0.31	-0.63	-0.46	-0.69	-1.39	-1.23	-1.68
	(0.120)	(0.118)	(0.112)	(0.782)	(0.795)	(0.754)	(0.532)	(0.612)	0.493	(0.164)	(0.220)	0.092
**Sargan test (p-value)**	(0.942)	(0.991)	(0.954)	(0.741)	(0.739)	(0.701)	(0.622)	(0.572)	(0.627)	(0.415)	(0.381)	(0.489)
**No. Obs**	1311	1311	1368	598	598	598	46	46	46	184	184	184
**East Asia and pacific**					**South Asia**				**Latin America and Caribbean**			
**Models**	**MD1**	**MD2**	**MD3**		**MD1**	**MD2**		**MD3**	**MD1**	**MD2**		**MD3**
**C**	-17.00***	-15.94***	-18.01***		.230	-2.13***		.3352	1.340***	1.629***		1.319***
	(0.00)	(0.00)	(0.00)		0.440	(0.00)		(0.282)	(0.00)	(0.00)		(0.00)
**CO2**_**-1**_	.997***	.995***	.997***		.971***	.973***		.970***	.946***	.946***		.946***
	(0.00)	(0.00)	(0.00)		(0.00)	(0.00)		(0.00)	(0.00)	(0.00)		(0.00)
**GDP**	.00016**	.00014**	.00016**		.00051**	.00050***		.00051***	.00014***	.00014***		.00014****
	(0.00)	(0.00)	(0.00)		(0.00)	(0.00)		(0.00)	(0.00)	(0.00)		(0.00)
**GDPS**	-.00000021***	-.00000018***	-.00000021***		-.00000013***	-.0000002***		-.00000013***	-.00000011***	-.00000012***		-.000000114***
	(0.00)	(0.00)	(0.00)		(0.00)	(0.00)		(0.00)	(0.00)	(0.00)		(0.00)
**BECS**	2.083***	----	----		21.18***	----		----	1.478***	----		----
	(0.00)	----	----		(0.00)	----		----	(0.00)	----		----
**WECS**	----	-1.896***	----		----	7.826***		----	----	2.515***		----
	----	(0.00)	----		----	(0.00)		----	----	(0.00)		----
**TECS**	----	----	1.916		----	----		25.78	----	----		1.33***
	----	----	(0.00)		----	----		(0.00)	----	----		(0.00)
**TO**	.771***	.614***	1.01***		.686***	.762***		.641***	.258***	.286***		.270***
	(0.00)	(0.00)	(0.00)		(0.00)	(0.00)		(0.00)	(0.00)	(0.00)		(0.00)
**PRG**	-.036**	-.067***	-.086***		.0020	-.015		-.020	-.116***	-.121***		-.1179***
	(0.01)	(0.00)	(0.00)		(0.93)	0.635		0.478	(0.00)	(0.00)		(0.00)
**AR (1)**	-1.76	-1.49	-1.70		-3.02	-3.20		-2.94	-6.04	-7.25		-6.12
	(0.078)	(0.135)	(0.089)		(0.003)	(0.001)		(0.003)	0.000	(0.000)		(0.000)
**AR (2)**	-0.73	-1.49	-0.34		-1.93	-0.94		-1.13	-0.46	-1.29		-0.52
	(0.464)	(0.136)	(0.730)		(0.053)	(0.349)		(0.258)	(0.646)	(0.196)		(0.600)
**Sargan test (p-value)**	(0.348)	(0.313)	(0.346)		(0.416)	(0.450)		(0.424)	(0.830)	(0.847)		(0.835)
**No. Obs**	230	230	230		69	69		69	192	192		192

See note under Table 3.

Recently, the importance of renewable energy to safeguard the environment has risen. However, the study also found inequalities in renewable energy consumption. The impact of renewable energy consumption inequality on the environment is reported in Table 7. The renewables consumption inequalities between the regions have a positive impact on the world, Europe & Central Asia, South Asia, East Asia & Pacific, North America environment. However, due to less access to renewable energy sources, the energy inequality between the Latin America & Caribbean region has a negative and insignificant impact on the environment. The renewable consumption inequality within the regions is positive in the world, Europe & Central Asia and Latin America & Caribbean. While, it is negative in the case of North America, East Asia & Pacific, and South Asia. The total renewable energy consumption inequality has a positive impact on the environment in all regions except world and East Asia & pacific. In renewable energy consumption, trade has a negative impact on the environment in the case of Europe & Central Asia. In contrast, it increases the environmental pollution in the world, South Asia, East Asia & Pacific, North America, and Latin America & Caribbean regions. The positive impact of trade on the environment is consistent with previous studies [24, 25]. However, trade remained negative in North America region. Again we find the negative impact of political regimes on the environment. Moreover, the results of GDP and square of GDP indorsed our previous results and confirm the inverted U-Shaped EKC curve in the countries.

**Table 7:** Results of renewable energy inequality.

	**World**			**Europe and Central Asia**			**North America**		
**Models**	**MD1**	**MD2**	**MD3**	**MD1**	**MD2**	**MD3**	**MD1**	**MD2**	**MD3**
**C**	1.493***	-3.354***	-3.873***	-16.32***	-16.66***	-15.11***	-10.97***	-10.48	-11.47
	(0.00)	(0.00)	(0.00)	(0.00)	(0.00)	(0.00)	(0.00)	0.862	(0.838)
**CO2**_**-1**_	.985***	.986***	.986***	.639***	.648***	.637***	.836***	.879***	.835***
	(0.00)	(0.00)	(0.00)	(0.00)	(0.00)	(0.00)	(0.00)	(0.00)	(0.00)
**GDP**	.00024***	.00014***	.00014***	.000045***	.000045***	.000036**	.00025***	.00015***	.00022***
	(0.00)	(0.00)	(0.00)	(0.00)	(0.00)	(0.014)	(0.00)	(0.00)	(0.00)
**GDPS**	-.00000031***	-.00000013***	-.00000013***	-.00000063***	-.00000068***	-.00000048***	-.00000027***	-.00000018***	-.00000023***
	(0.00)	(0.00)	(0.00)	(0.00)	(0.00)	(0.017)	(0.00)	(0.00)	(0.000
**BECS**	.3665***	----	----	.856***	----	----	4.742***	----	----
	(0.00)	----	----	(0.00)	----	----	(0.00)	----	----
**WECS**	----	.108***	----	----	.297**	----	----	1.081**	----
	----	(0.00)	----	----	(0.013)	----	----	(0.00)	----
**TECS**	----	----	-.228***	----	----	.614**	----	----	7.299***
	----	----	(0.00)	----	----	(0.017)	----	----	(0.00)
**TO**	1.096***	.642***	.512***	.767***	.790***	.580**	-.065***	-.0793***	-.054***
	(0.00)	(0.00)	(0.00)	(0.00)	(0.00)	(0.01)	(0.00)	(0.00)	(0.00)
**PRG**	-1.852***	-.617***	-.616***	-.228***	-.519***	-.025***	-1.826***	-1.256***	-1.473***
	(0.00)	(0.00)	(0.00)	(0.00)	(0.00)	(0.00)	(0.00)	(0.00)	(0.00)
**AR (1)**	-16.75	-16.87	-14.81	-6.79	-6.82	-6.75	-0.61	-0.28	-0.62
	(0.000)	(0.000)	(0.000)	(0.000)	(0.000)	(0.00)	(0.543)	(0.777)	(0.536)
**AR (2)**	-1.49	1.45	1.60	0.26	181.99	0.32	-0.56	-0.45	-0.56
	(0.136)	(0.148)	(0.110)	(0.796)	(0.270)	(0.753)	(0.574)	(0.656)	(0.574)
**Sargan test (p-value)**	(0.797)	(0.739)	(0.974)	(0.628)	(0.688)	(0.703)	(0.546)	(0.608)	(0.545)
**No. Obs**	1311	1311	1311	598	598	598	46	46	46
	**East Asia and pacific**			**South Asia**			**Latin America and Caribbean**		
**Models**	MD1	MD2	MD3	MD1	MD2	MD3	MD1	MD2	MD3
**C**	-16.74***	-16.80***	-16.74***	-2.318***	.518***	-1.89***	1.593***	.981***	1.44***
	(0.00)	(0.00)	(0.00)	(0.00)	0.071	(0.00)	(0.00)	(0.00)	(0.00)
**CO2**_**-1**_	.800***	.863***	.809***	.967***	.962***	.962***	.946***	.949***	.946***
	(0.00)	(0.00)	(0.00)	(0.00)	(0.00)	(0.00)	(0.00)	(0.00)	(0.00)
**GDP**	.00015***	.00016***	.00015***	.00054***	.00054***	.00053***	.00014***	.00014***	.00015***
	(0.00)	(0.00)	(0.00)	(0.00)	(0.00)	(0.00)	(0.00)	(0.00)	(0.00)
**GDPS**	-.00000020***	-.00000021***	-.00000020***	-.00000014***	-.00000014***	-.00000014***	-.00000012***	-.00000012***	-.00000013***
	(0.00)	(0.00)	(0.00)	(0.00)	(0.00)	(0.00)	(0.00)	(0.00)	(0.00)
**BECS**	3.23***	----	----	2.20***	----	----	-.4671	----	----
	(0.00)	----	----	(0.00)	----	----	(0.649)	----	----
**WECS**	----	-.232***	----	----	-3.83***	----	----	1.118***	----
	----	(0.00)	----	----	(0.00)	----	----	(0.00)	----
**TECS**	----	----	-1.162***	----	----	10.01***	----	----	1.66
	----	----	(0.00)	----	----	(0.00)	----	----	(0.16)
**TO**	.733***	.721***	.672***	.435***	1.067***	.624***	.170	.186***	.193**
	(0.00)	(0.00)	(0.00)	(0.00)	(0.00)	(0.00)	(0.012)	(0.00)	(0.01)
**PRG**	-.121***	-.082***	-.1137***	-.020	-.044*	-.0030	-.109***	.0038	-.0102
	(0.00)	(0.00)	(0.00)	(0.42)	(0.07)	(0.89)	(0.00)	(0.89)	(0.759)
**AR (1)**	-0.74	-0.29	-0.32	-8.82	-3.67	-23.90	-5.77	-5.96	-5.78
	(0.457)	(0.772)	(0.751)	(0.000)	(0.000)	(0.000)	(0.000)	(0.000)	(0.000)
**AR (2)**	-0.73	-0.63	-0.09	-1.47	-1.05	-1.56	0.81	0.81	0.78
	(0.462)	(0.526)	(0.927)	(0.142)	(0.296)	(0.118)	(0.417)	(0.417)	(0.817)
**Sargan test (p-value)**	(0.357)	(0.583)	(0.646)	(0.464)	(0.477)	(0.459)	(0.810)	(0.833)	(0.810)
**No. Obs**	230	230	230	69	69	69	184	184	184

See note under Table 3.

By considering the environmental Kuznets curve, the environmental situation can be alarming at the early stage of the development as the country starts trade its income and production level grows. However, at this stage, countries have more pollution because of less access to cleaner energy resources. Additionally, economies are mostly dependent on obsolete methods of production that consume more energy and emit more carbon. So, trade impact possibly is positive. In addition, trade does not enhance environmental quality if a nation produces high-emission products. Secondly, developing nations are causing more pollution by the lax rules and regulations laid down in free trade agreements [20, 54, 55]. Under the race to the bottom hypothesis, trade may have hazardous effects on the environment especially in the case of developing countries [20]. Yasmeen et al [29] also find that the trade impact on eight air pollution indicators is positive at first stage of the development but it leads to improving the environmental quality in the second phase of the development. In this context, our results are in line with the extant literature. In renewable energy, Europe & Central Asian region seems to improve the environment by trading as the coefficient sign is negative.

## 5. Conclusion

The world is experiencing rapid growth in energy consumption due to economic expansion and population growth. Thus, keeping in view the worth of energy in the growth process and trade sector, this study finds inequalities in oil, coal, natural gas, hydropower, and renewables. Energy inequalities by renewable and non-renewable sources provide insightful information for policy development. The inequalities are calculated by applying “Theil’s cross-entropy” that shed light on inter- and intra- energy consumption among six regions of the world.

North America has the highest oil consumption inequality between the regions while, East Asia & Pacific region has the highest index value within the region. Most importantly, all regions are experiencing a declining trend in oil consumption inequality. However, inequality in coal consumption is decreasing between the North American. Inequality in coal consumption is increasing in East Asia & Pacific regions. This increasing trend in inequalities is possibly due to China’s economic expansion. Regardless of the regional situation, the overall world is experiencing decreasing inequality in coal consumption. Europe & Central Asia, and North America are two major regions that have the highest inequality in natural gas consumption between the regions. While, East Asia & pacific have the highest level of energy consumption inequality within regions. Moreover, there is a downward tendency in natural gas consumption inequality between and within regions. Inequality is decreasing in hydropower consumption between regions; however such a trend has not loomed within the regions. North America has large disparities between the regions that declined over the time. Within regions, North America and Europe & Central Asia have a higher-level of inequality in hydro-energy consumption, which remains almost sustained over the time except in 2009. In contrast, total energy consumption inequality is decreasing over the time. In East Asia & Pacific, however, there is a rising trend in total inequality in hydro consumption. Europe & Central Asia, and East Asia & Pacific have major inequalities in renewable consumption within regions. However, there is a decreasing trend over the time. The inequality gap is also diminishing between North American regions for renewable energy sources. In total renewable energy consumption inequality, we find a decreasing pattern for all regions. By using the GMM method, we discover that inequalities in energy consumption have a positive effect on the quality of the environment. Trade is considered the engine of growth and development in the economy. Trade has a positive impact on environmental quality. The study also shows that the democratic political regime can be advantageous to improve environmental quality. Despite this, the study results validate the inverted U-shaped impact on environmental quality.

Although the study found the declining trend in energy inequality, the mechanism of the energy sector still needs to be improved. The primary cause of energy inequalities and carbon emissions is the growth in power demand. Switching to renewable energy is not the only way to decarbonize. Therefore, unlike just renewable energy, advanced technology needs to be adopted that consumes less energy and fewer emissions. Trade can help to reduce energy inequalities by opening the doors to more equal opportunities for developing countries towards energy sources. Developing economies, however, are not good at shaping the effect of trade on the environment due to emission-intensive goods. Therefore, its compositional impact (engagement to products) must be revised in order to enjoy trade-led growth with the green environment. Institutional reforms are also important for improving trade and energy efficiency. To balanced energy consumption and strong institutions can actively regulate inequalities within regions. In addition, the identification of inequalities in distinct sources of energy can provide policymakers with helpful guidance on energy consumption and sustainable growth. Though the present study found considerable variations in energy inequality among the regions, however, overall inequality in oil consumption has decreased over the time. This declining trend in oil energy consumption inequalities shows equal access to oil resources. Overall this study implies that countries should take necessary actions to reduce energy inequalities within and between the regions. Specialization in production through trade can also be an option for improvement in the environment. The study has limitation as each country has different resource endowments, different climates, different economic developments, different industrial structures, and different levels of technology. The consumption of energy per capita is naturally different thus the analysis is also conducted at regional level to minimize the country specific effects as these differences are comparatively low at regional level.

## Supporting information

S1 Appendix (DOCX)
