# Supplementary material for: Inequalities by energy sources: An assessment of environmental quality
Source: PLoS One. 2020 Mar 20;15(3):e0230503. doi: 10.1371/journal.pone.0230503 (PMC7083316; doi:10.1371/journal.pone.0230503)
Supplement: S1 Appendix — (DOCX) [file pone.0230503.s001.docx]

**Table A1 Countries list**

| **regions** | **countries** | | | | | | | |
| --- | --- | --- | --- | --- | --- | --- | --- | --- |
| Latin America and Caribbean | Argentina | Brazil | Chile | Colombia | Ecuador | Mexico | Peru | Venezuela |
| East Asia and pacific | Australia | japan | New Zealand | China | Indonesia | Malaysia | Philippines | Singapore |
|  | Thailand | Vietnam |  |  |  |  |  |  |
| Europe  and  Central Asia | Austria | Belgium | Czech Republic | Finland | France | Germany | Greece | Hungary |
|  | Italy | Netherlands | Norway | Poland | Portugal | Romania | Spain | Sweden |
|  | Switzerland | United Kingdom | Azerbaijan | Belarus | Kazakhstan | Russian Federation | Turkey | Turkmenistan |
|  | Ukraine | Uzbekistan |  |  |  |  |  |  |
| North America | Canada | United stats |  |  |  |  |  |  |
| Middle East and North Africa | Algeria | Egypt | Iran | Israel | Kuwait | Qatar | Saudi Arabia | United Arab Emirates |
| South Asia | Bangladesh | India | Pakistan |  |  |  |  |  |
